# Reducing mental health stigma in the workplace: a mixed-method analysis of a quasi-experimental trial and the contextual role of personal values

**DOI:** 10.3389/fpubh.2026.1758132

**Published:** 2026-04-17

**Authors:** Elena Stoll, Emily Nething, Sara Marie Uhlig, Josefine Retka, Karoline von Finckenstein, Christin Schwiebert, Samuel Tomczyk

**Affiliations:** 1 Institute of Psychology, Department of Health and Prevention, University of Greifswald, Greifswald, Germany; 2 Institute of Medical Psychology and Medical Sociology, University Medicine Rostock, Rostock, Germany

**Keywords:** mental illness stigma/mental health stigma, mixed-methods design, personal values, resilience, workplace intervention

## Abstract

**Background:**

Workplace mental illness stigma is associated with negative consequences for individual, social, and structural (e.g., employment) aspects. Thus, multilevel interventions such as the Canadian program *The Working Mind* (TWM) are needed to address all aspects of stigma simultaneously. Regarding stigma reduction, few studies have examined the potential to also increase mental health literacy and resilience or addressed personal values.

**Aim:**

In this first international adaptation of TWM, we culturally adapted and evaluated TWM in the context of a German university regarding stigma, mental health literacy and resilience, while examining the role of values.

**Methods:**

The study followed a quasi-experimental, sequential explanative mixed-methods research design (QUAN → qual) with assessments before, after the intervention and at 6-month follow-up. The sample consisted of managers and employees (intervention group: *N* = 69; control group: *N* = 49).

**Results:**

The intervention group showed significantly higher mental health literacy post-intervention compared to the control group after adjusting for baseline values, with a large effect size. Other effects were mainly small- to medium-sized but non-significant. Personal values of self-transcendence did not moderate the effect on mental illness stigma. Qualitative results showed an improvement on resilience for managers and employees and replicate the increase in mental health literacy.

**Discussion:**

Overall, we observed positive effects and trends of TWM regarding stigma, mental health literacy, and resilience. Possibly due to power limitations, baseline-adjusted group differences in stigma and resilience did not reach statistical significance. Value-based measures may explain inter-individual differences. Replication studies are needed to examine the observed effects in larger samples.

## 1 Introduction

According to the WHO, about 15% of working-age adults worldwide live with a mental disorder, primarily depression and anxiety, which cost the global economy US $1 trillion each year due to reduced productivity alone (1).

The stigma associated with mental illness is an additional burden for those affected, as it can increase the risk to career losses and precarious employment (2), the avoidance of help-seeking and the exacerbation of mental health symptoms (3, 4). In recent years, awareness of the need for interventions to destigmatize mental illness, also in the workplace, has increased (2, 5).

Mental illness stigma can be seen as a process of building negative stereotypes, separation of an “in-” and an “out-” group (i.e., people living with mental illness) and discrimination against the “out”- group (i.e., people living with mental illness) (6, 7). According to Pescosolido and Martin (6), public stigma is defined as “stereotypes, prejudice, and discrimination endorsed by the general population” and self-stigma is referred to as the “internalized acceptance of stereotypes and prejudice.” Addressing workplace mental illness stigma in this manuscript, we refer primarily to public stigma, but self-stigma can also play a role at the workplace, for example, in (non-)disclosure of mental health problems or help-seeking (8). With mental illness and mental health regarded as a continuum, we treat the terms “mental illness stigma” and “mental health stigma” as synonymous.

In their systematic review, Tóth et al. (2) found various multimodal programs using different techniques such as psychoeducation, skills training and lived experience vignettes/videos to address workplace mental illness stigma. Most of the quantitatively evaluated interventions published between 2010 and 2021 found a significant decrease in stigmatizing attitudes related to mental health, some of them even after a 6-month follow-up. Furthermore, some interventions resulted in changes in other psychological measures, for example, increased mental health literacy, increased resilience or intention to seek help.

Tóth et al. (2) reported significant effects of several manualized programs, such as The Working Mind (TWM) (9) or Mental Health First Aid (MHFA) (10–13). Benefits of TWM compared to other promising interventions are the shorter duration of the intervention (maximum of 4 h for employees or 8 h for managers, e.g., compared to 6–12 h of MHFA) and the explicit focus on employees and managers. TWM not only focuses on mental health (literacy) but also addresses the return-to-work (RTW) process for managers and fosters their communication skills. Previous research points to benefits of RTW interventions even from an economical point of view (14). As TWM fosters resilience (9), one could assume that according to Brown et al. (15), changes could occur on different levels: the intrapersonal, interpersonal, and organizational. Firstly, employees and managers could benefit personally (by gaining new coping options). Secondly, resilience may also develop interpersonally, in the team context, for example, because communication skills are improved and there is more openness toward mental health issues. There is some evidence that anti-stigma interventions may positively impact openness toward mental health issues and help-seeking behavior (16), although these relationships appear to be complex (17). Thirdly, TWM could foster organizational resilience as described by Brown et al. (15), for example, by improving managers' understanding of the RTW-process.

However, most of the previous research stems from the US, UK, Australia, or Canada—as is also the case for TWM –, and there is little understanding which contextual factors could help to reduce stigma in the workplace and how anti-stigma programs work in different cultural contexts (2, 11, 12, 18).

One aspect that might be relevant in this area, but which, to our knowledge, has not yet been assessed in anti-stigma interventions, is personal values. Yang et al. (19) argue that mental health stigma affects “lived values in everyday life activities” [(19), p. 1532] or “what matters most,” differing between cultures and individuals. The authors call for multiple perspectives and measures in assessing mental illness stigma. They argue that viewing the stigma processes as a moral experience is crucial for a better understanding. Shalom Schwartz' Theory of Basic Values (20, 21) is, to date, the most elaborated theory of personal values with cross-cultural evidence. Values are “trans-situational goals, varying in importance, that serve as guiding principles in the life of a person or group” [(20), p. 664]. Schwartz (21) proposed a circular motivational continuum of ten basic values with four superordinate values: (1) self-transcendence values consisting of universalism (“understanding, appreciation, tolerance and protection for the welfare of all people and for nature”) and benevolence (“caring for ingroup members”); (2) openness to change values including self-direction (“independent thought and action in choosing, creating, exploring”), hedonism (“pleasure and sensuous gratification for oneself”) and stimulation (“excitement, novelty, and challenge in life”); (3) self-enhancement containing achievement (“personal success through demonstrating competence according to social standards”) and power (“social status and prestige, control or dominance over people and resources”); and finally, (4) conservation values consisting of security (“safety, harmony, and stability of society, of relationships, and of self”), tradition (“maintaining cultural and religious traditions”) and conformity (“avoiding upsetting others and compliance with social norms”) (20). Personal values can influence attitudes and behavior (22, 23) and are context dependent (22).

The idea that personal values are linked to moral social attitudes gains support by the work of Boer and Fischer (22). They showed that personal values according to Schwartz' theory (20) are linked to moral social attitudes; for example, the values of self-transcendence are linked to moral foundations of care, which involves caring about others. There is some evidence that higher self-transcendence values are connected to reduced desire for social distance (24) and to positive affect toward people living with mental illness (25). Conservation values on the other hand are assumed to be connected to higher public mental health stigma attitudes (24, 26). Most of the studies focusing on the effects of values on (de-) stigmatization of mental illness concentrate on intercultural value differences (27–31), disregarding cultural differences in personal values within the same culture (26). Schomerus and Angermeyer (26) discuss the relationship of stigma in conservative and liberal milieus, and call for more research on mental illness stigma in the latter. Finally, Rieckhof et al. (32) have developed a value-sensitive stigma questionnaire (VASI), based on Shalom Schwartz' theory of human values (21), designed to measure mental health stigma in liberal cohorts.

In this study, we explore the impact of a workplace anti-stigma intervention (The Working Mind) in the German higher education context while considering the role of values. Universities are relevant for workplace mental health research because they offer a mix of scientific and non-scientific (administrative or technical) staff and international employees and students, i.e., future employees. Further, academic staff face several stressors which increase the likelihood for mental health problems (33–35). In a university setting, values between scientific and non-scientific staff may differ and thus, moderate the outcome of the intervention. The first reason is that scientific staff generally have higher education levels than non-scientific staff. There is evidence from research that higher educational levels increase the probability that a person holds more libertarian rather than authoritarian values (36), so this might affect mental health stigma, as well. Second, looking at stigma as “a moral experience” affecting “what is most at stake in a local world” [(19), p. 1525], the groups could differ on what they value most. According to Kinman (37), scientific staff is more likely to show overcommitment at work, which means “excessive striving in combination with a strong desire of being approved and esteemed” [(37), p. 185]. Due to this motivation, being confronted with mental health issues may affect the image of a “successful scientist,” due to fears of rejection or disapproval by meaningful others (38). Therefore, it may be associated with higher stigmatization of mental illness and lower interest in dealing with mental health issues.

We consider this study as explorative, as it is the first evaluation of “The Working Mind” in higher education within a different cultural (Germany) and working (higher education) context and under a quasi-experimental setting with a control group: our main study aim is to examine the effects of the adapted intervention on public mental health stigma, openness for psychological problems, help-seeking intentions, resilience and mental health literacy in order to generate hypotheses about the effectiveness of the program in other cultural contexts. As a second aim, we investigate how values might play a role in our adapted intervention, given that they can influence attitudes and behavior and that their manifestation is also shaped by the cultural context. More specifically, a moderating effect of self-transcendence values on stigmatizing attitudes might be plausible, with higher self-transcendence values leading to more reduction in mental illness stigma. Relatedly, it is of interest to explore whether, and if so which, values are promoted by the intervention TWM, as values can influence changes in stigmatizing attitudes (23).

## 2 Methods

### 2.1 Trial design and deviations from the study protocol

**Figure 1:**
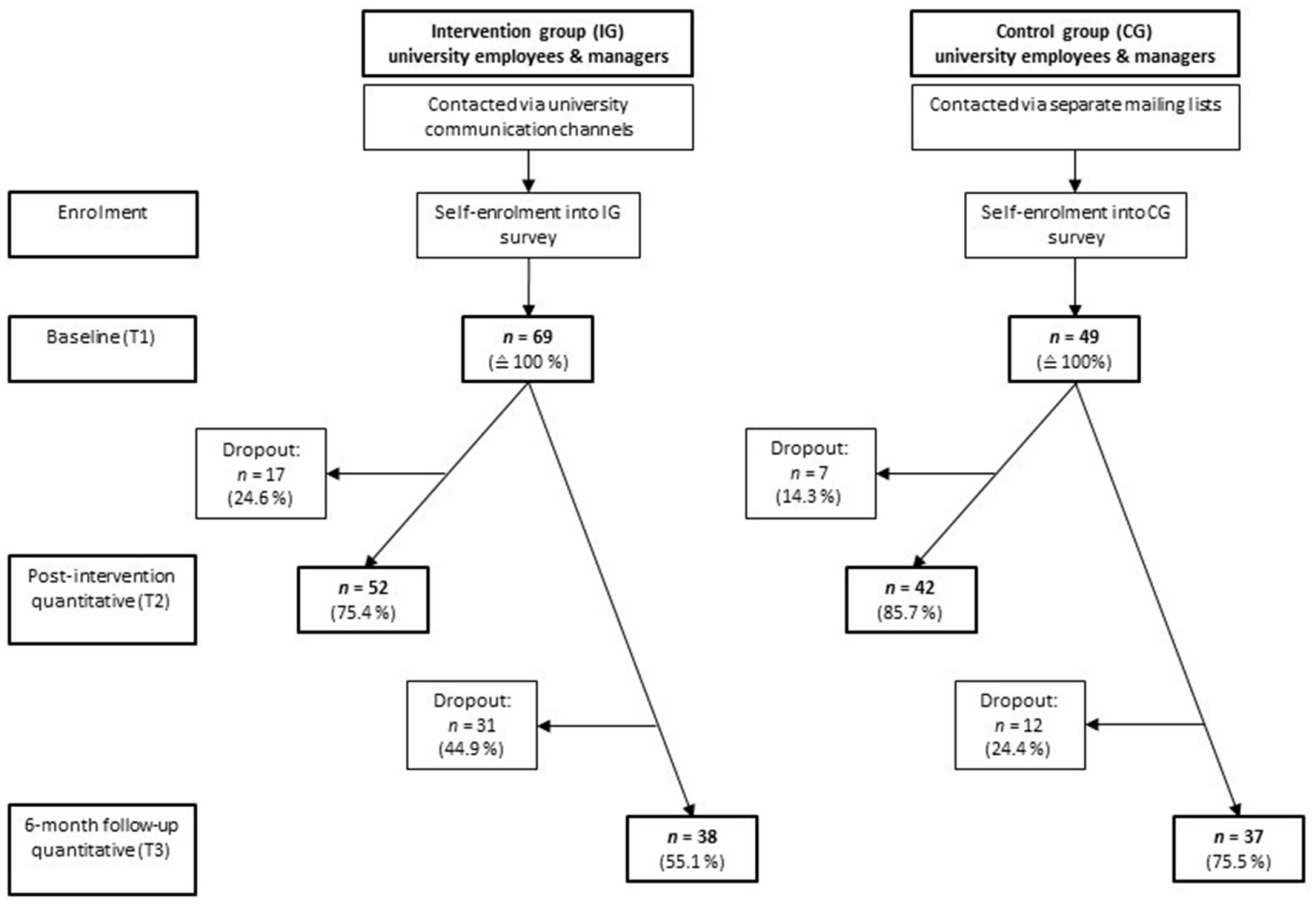
Participant flow. There were a few survey participants who only took part in the T2 and/or T3 assessments: *n* = 1 in the intervention group at T2 and T3, and *n* = 4 in the control group at T3. These participants had to be excluded due to the nature of our analyses, as they require T1 data.

To examine the efficacy of TWM in a German higher education context and to understand what works for whom, we implemented a sequential explanative mixed methods design (QUAN → qual) comprising two steps: (1) quantitative longitudinal surveys in a quasi-experimental set-up, and (2) a qualitative post-intervention focus group or interview with employees and managers (see Figure 1). As described in more detail in the study protocol (39), the quantitative part of the study investigated the program's efficacy. The qualitative part aimed to elaborate and expand on the quantitative findings, with a particular focus on the values promoted by TWM. The quantitative part comprised three timepoints: the T1 baseline questionnaire was assessed pre-intervention, the T2 questionnaire was assessed post-intervention, and the T3 follow-up questionnaire was assessed 6 months after T2. For a more detailed description of our decision to utilize a mixed-methods design and the study planning and procedures, see Nething et al. (39).

The program's target groups, i.e., employees and managers, have been involved in the entire research process, in line with the GRIPP2 recommendations (40) (see Supplementary Table S1). We sought and incorporated the target groups' feedback on the research questions, the culturally adapted program materials, and the culturally adapted key outcome measurement instrument (Opening Minds Scale for Workplace Attitudes; OMS-WA; see section 2.4). Members of the target groups also participated in recruiting participants by encouraging their colleagues to take part in the program. Beyond the participatory elements in specific project phases, the study was affiliated with an internal university advisory board. The board consisted of representations of all target groups and reflected on the research process, with a particular emphasis on sustainable implementation. More details can be found in the study protocol (39). Study protocol and supplementary material are available at OSF (link at the end of the manuscript).

Due to low registration numbers, we conducted one focus group with employees and additionally, interviews with employees and managers, offering them the same incentives (€30 vouchers). In the analysis, mental health literacy is treated as an outcome and not as a mediator given the small sample size and effect sizes for stigma outcomes as well as the correspondingly low power.

Due to group differences regarding baseline values of the outcomes and sociodemographic characteristics, we had to change our statistical analysis strategy with regard to our initial plans in the study protocol. As described in the study protocol (39), we initially planned to test the main effect hypotheses using 2 × 3 ANOVAs, with group (intervention, control) as the between-subjects factor, and time (T1, T2, and T3) as the within-subjects factor, and the group × time interaction representing the intervention effect. However, to account for the aforementioned differences, we instead conducted baseline-adjusted ANCOVAs comparing the groups at T2 and T3, respectively. A more detailed description of statistical procedure is provided in section 2.4. For employee T3 and data from managers at all timepoints, we were not able to reach as many participants as we had planned for initially (calculations were based on an *a priori* power analysis for the 2 × 3 ANOVAs, assuming a medium effect size): *n* = 50 employees and *n* = 50 managers (39). Therefore, we decided to combine the employee and manager sample (since the managers taking part in our study are university employees, and some employees are also managers, thus blurring the lines). Further, since conservation values, initially planned to be tested as a moderator, turned out to differ meaningfully between groups, we introduced them as a confounder instead.

### 2.2 Participants, procedure, and intervention

To participate in the study, participants had to be at least 18 years old and work at a German higher education institution. Both employees with and without personnel responsibility were able to take part in the study; the former will be referred to as managers in the following. Participants were recruited via self-selection from February 2024 to June 2024, with last follow-up data acquisition in January 2025. Intervention participants were recruited via flyers and via the university's communication channels, control group participants via mailing lists (39). All participants were granted vouchers for their participation (€10 for completed online surveys).

A few days before the intervention began, participants in both the intervention and control groups were invited to take part in the quantitative baseline survey (T1). Subsequently, the intervention group took part in the TWM workshop and was informed about the opportunity to participate in post-intervention focus groups. Shortly after the intervention, participants in both groups were contacted again to complete the quantitative post-intervention survey (T2), and intervention group participants who had expressed interest were additionally contacted regarding the focus groups. These took place approximately 8 weeks after the intervention. Approximately 6 months after the intervention, participants in both groups were contacted again regarding the follow-up survey (T3).

The project is based on the guidelines of Good Scientific Practice of the German Research Foundation and Good Clinical Practice of the German Society for Epidemiology. For the implementation of the empirical studies in the project, approval was obtained from the local ethics committee (Ethics Committee of University Medicine Greifswald; BB 098/23). Participants had to provide written consent before taking part in a focus group or interview. As for the online survey, participants had to give their consent by agreeing to an online data protection form before they could start completing the survey.

The intervention, the workshop TWM, was delivered by the second and the last author who took part in a 1-week training course for TWM facilitators to ensure fidelity in implementation. The translation and adaptation of intervention materials followed a co-creation principle with regular meetings with the original developers to ensure quality. Before conducting the first workshop, the trainers discussed the materials and implementation and prepared all sessions and approaches together to ensure consistent delivery. For these reasons, an inter-trainer consistency was not assessed.

The workshop lasted from 4 h (employees) to 8 h (managers). It was provided in small face-to-face groups of up to 15 people on the premises of our university. Five workshops for employees and two workshops for managers were delivered between February and June 2024. To create a warm and appreciative atmosphere, participants were offered non-alcoholic drinks and a cold buffet.

TWM is a multilevel intervention combing psychoeducation (e.g., on stigma, stress and resilience), video testimonials from persons with lived experience and exercises to build resilience as well as group discussions (9, 39). It consists of three modules for employees and four modules for managers (see Supplementary Table S2 for details). The modules concentrate on mental illness and stigma, the Mental Health Continuum Tool, self-care and building resilience. Managers additionally learn about communication skills, healthy working environments and accompanying employees in returning to the workplace after a leave due to ill mental health. The information was given via PowerPoint presentations and supplementary printed material (e.g., handbook, pocket card).

### 2.3 Study hypotheses and outcome measures

Testing TWM in the context of a medium-sized German university, we expected small to medium size effects of TWM on reduced public mental health stigma (H1), increased openness for psychological problems (H2), help-seeking intentions (H3), resilience (H4) and mental health literacy (H5). Furthermore, we assumed moderator hypotheses for personal values, namely self-transcendence (H6quan). Similarly, we explored whether, and if so which, values are promoted by TWM (F6qual). We investigated hypotheses H1–H5 using both quantitative and qualitative data.

Information on the quantitative measurement instruments and the reasons for their selection are described in detail in the study protocol (39), the following overview is based on this study protocol. For the qualitative part of the study, semi-structured focus group and interview guides were used, as described in and published along with the study protocol (39).

**(H1) Mental health stigma** was measured in several facets using the following questionnaires: *Workplace specific stigma* was assessed with the German version of the Opening Minds Scale—Workplace Attitudes (OMS-WA) in the employee version (9, 41). It comprises 22 items, such as “People/employees with a mental illness could snap out of it if they wanted to.” Participants are asked to rate the items on a 5-point Likert scale from 1 = “strongly disagree” to 5 = “strongly agree.” The items form five subscales: Avoidance/Social Distance, Dangerousness/Unpredictability, Work -Related Beliefs/Competency, Helping Behaviors, Perceptions of Responsibility. We translated the OMS-WA questionnaire into German using a forward-back translation procedure, consulting an expert panel, and pre-testing it with the target group. Reported internal consistency is good for the employee version (α = 0.86) and the questionnaire shows good construct validity (41).

*Public mental illness stigma* (context-unspecific) was assessed with the German version of the Self-Stigma of Mental Illness Scale—Short Form (SSMIS-SF) (42, 43) *Agreement* subscale. This subscale captures public stigma (which represents a necessary component of self-stigma). Participants are asked to rate the five items such as “I think most persons with mental illness are dangerous” on a 5-point Likert scale from 1 = “strongly disagree” to 5 = “strongly agree.” Studies across multiple clinical samples and languages indicate good construct validity of the scale, internal consistencies for the *Agreement* subscale are acceptable (72 ≤ α ≤ 0.79) (42).

*Value-sensitive mental illness stigma* was assessed with the Value-based Stigma Inventory (VASI) (32), a German questionnaire. Participants are asked to rate the 15 items such as “It damages my reputation if a mental illness becomes known in my family” on a 5-point Likert scale from 1 = “strongly disagree” to 5 = “strongly agree.” The items form five subscales: Self-Realization, Personal Enrichment, Reputation, Meritocratic Values, and Security. Rieckhof et al. (32) report good internal consistency (α = 0.88), and good convergent and construct validity of their questionnaire.

*Public stigma of seeking help* was assessed with the German version of the Stigma Scale for Receiving Psychological Help (SSRPH) (44). Participants are asked to rate the five items such as “Seeing a psychologist for emotional or interpersonal problems carries social stigma” on a 4-point Likert-scale from 0 = “strongly disagree” to 3 = “strongly agree.” Internal consistency for the scale is good (α = 0.81) and the original study indicated good construct validity [62]; however, information on its validity remains sparse.

*Self-stigma of seeking help* was assessed with the German version of the Self-Stigma of Seeking Help (SSOSH) (44, 45). Participants are asked to rate 10 items such as “I would feel worse about myself if I could not solve my own problems” on a 5-point Likert scale from 1 = “strongly disagree” to 5 = “strongly agree.” Internal consistency for the scale is acceptable to good (0.80 ≤ α ≤ 0.84). Information on the validity of the German version is not yet available, but the original version has proven valid in terms of construct, criterion, and predictive validity.

**(H2) Openness to mental health problems** was assessed at timepoints 1–3, using the German version of the Inventory of Attitudes to Seeking Mental Health Services (IASMHS) (46) *Psychological Openness* subscale. The *Psychological Openness* subscale is reverse-coded, meaning that higher scores indicate less openness. Participants are asked to rate the eight items such as “There are certain problems which should not be discussed outside of one's immediate family” on a 5-point Likert scale from 0 = “disagree” to 4 = “agree.” Internal consistency for the *Psychological Openness* subscale is acceptable (α = 0.70). Convergent validity for the original scale could be demonstrated by Mackenzie et al. (47).

**(H3) Willingness to seek help** was measured at timepoints 1–3 by asking participants to rate how likely it would be for them to use different support offers, using a 6-point Likert scale from 1 = “not at all likely” to 7 = “very likely.” They were provided with a list of 17 support offers discussed during the intervention and asked to rate the likelihood for each of the offers. **Utilization of support offers** was measured at timepoint 3 by asking participants if they have used one or more support offers.

**(H4) Resilience** was measured at timepoints 1–3, using the German version of the Brief Resilience Scale (BRS) (48). Participants are asked to rate the 6 items such as “I tend to bounce back quickly after hard times” on a 5-point Likert scale from 1 = “strongly disagree” to 5 = “strongly agree.” Internal consistency for the scale is good (α = 0.85) (48). The BRS is moderately correlated with optimism and social support, indicating convergent validity (48).

**(H5) Mental health literacy** was assessed at timepoints 1–3, using the Mental Health Literacy Tool for the Workplace (MHL-W-G) (49). The MHL-W-G is reverse-coded, meaning that higher scores indicate lower mental health literacy. Depending on the item, participants are asked to rate the 35 items on either a 4-point Likert scale from 1 = “very unlikely/unhelpful” to 4 = “very likely/helpful” or a 5-point Likert scale from = “strongly disagree/definitely unwilling” to 5 “strongly agree/definitely willing.” They are, for instance, asked to rate statements such as “To what extent do you think it is likely that the diagnosis of Drug Dependence includes physical and psychological tolerance of the drug (i.e., require more of the drug to get the same effect)?” Internal consistency for the German version of the scale is good (0.88 < α < 0.92) (49). The scale shows good test-retest reliability and satisfactory convergent validity (49).

**(H6quan) Personal values** were measured at timepoints 1 and 3 with the Portrait Values Questionnaire 21 (PVQ 21, ESS) (50, 51), adapted in a gender-neutral form (32). The PVQ21 consists of 21 items presented in the form of short verbal portraits which describe a person's goals, aspirations or desires that point explicitly to each value, for example: “It's important to them to be rich.” The respondents are asked to rate the similarity on a scale from 1 = “very similar” to 6 = “not similar at all.” Reported internal consistencies are rather low, for example, self-transcendence scales (0.46 < α < 0.51 for Germany), but the authors argue, that despite this “the value indexes have shown substantial validity” (51). Personal values measured with PVQ21 have shown to be stable over a 3-year period, similar to personal traits (52).

The internal consistency of the scales in this study is reported in Table 1.

**Table 1:** Internal consistency of the scales in this study in the order of the hypotheses.

Hypotheses	Scale	Internal consistency (Cronbach's ***α***)		
		T1	T2	T3
(H1)	Opening Minds Scale – Workplace Attitudes (OMS-WA)	0.81	0.85	0.81
	Self-Stigma of Mental Illness Scale – Short Form (SSMIS-SF), *Agreement* subscale	0.70	0.84	0.75
	Value-based Stigma Inventory (VASI)	0.79	0.79	0.77
	Stigma Scale for Receiving Psychological Help (SSRPH)	0.73	0.71	0.78
	Self-Stigma of Seeking Help (SSOSH)	0.68	0.73	0.74
(H2)	Inventory of Attitudes to Seeking Mental Health Services (IASMHS) (46), *Psychological Openness* subscale	0.74	0.65	0.69
(H3)	Willingness to seek help items	0.89	0.91	0.87
(H4)	Brief Resilience Scale (BRS)	0.81	0.80	0.88
(H5)	Mental Health Literacy Tool for the Workplace (MHL-W-G)	0.91	0.91	0.92
(H6quan)	Portrait Values Questionnaire 21 (PVQ21), *Self-transcendence* subscale	0.55	–	0.66

Cronbach's α was calculated for timepoints T1–T3 for each scale, except for PVQ21 which was assessed at T1 and T3 only.

We aggregated individual participant data by calculating mean values or sum scores, depending on the instrument. Reverse-coded instruments were recoded to facilitate interpretation. Missing values were deleted pairwise.

### 2.4 Data collection, data management, and data analysis

Quantitative data was collected using the online platform SoSci Survey (53). Qualitative data was collected in one focus group and nine interviews using semi-structured interview guidelines. The focus groups/interviews were conducted by the research team (psychotherapist and psychologist with multiple years of experience in fieldwork), with the help of three additional research assistants (undergraduate and graduate students of Psychology). All interviewers were female and interested in destigmatizing mental illness.

The statistical analysis of the quantitative data was carried out using R, version 4.3.3 (54), and additive packages: tidyverse (55), magrittr (56), DT (57), car (58), psych, corrplot (59), sandwich (60, 61), and parameters (62).

There was a blinding for the control group, who were told they were taking part in a study designed to improve mental health conditions at the university. To reduce bias, persons who delivered the focus groups and the workshops were not the same.

For quantitative data, unit of analysis were groups (e.g., intervention vs. control group). Qualitative data was analyzed on the individual and group level (managers vs. employees).

Following Twisk et al. (63), we calculated ANCOVAs comparing the T2 (post-intervention) or T3 (6-month follow-up) scores, respectively, between the intervention and the control group, while controlling for the T1 (baseline) value of the outcome. Therefore, group differences at T2 reflect differences in post-intervention outcomes between participants with comparable baseline levels; correspondingly, group differences at T3 reflect differences in follow-up intervention outcomes between participants with comparable baseline levels. The grouping variable was coded 0 = intervention and 1 = control. Positive estimates thus indicate higher values in the control group compared to the intervention group, while negative estimates indicate lower values in the control group compared to the intervention group. While this approach does not model time as a within-subject factor as planned initially (see section 2.1), adjusting for baseline levels allows us to account for initial group differences and to examine group effects at T2 and T3 relative to the baseline levels.

In addition, we included relevant sociodemographic variables in which the groups differed as covariates (H1–H5), which is described in more detail in the section “Baseline equivalence.” For the moderator hypothesis regarding self-transcendence values (H6), we additionally included an interaction term group x self-transcendence values as planned.

The significance level is set to α = 0.05 for all statistical tests, except for tests for variance homogeneity (as part of the assumption tests), for which α = 0.10 is set since Levene's test assesses variance equality as the null hypothesis and a higher alpha level therefore increases sensitivity to violations of this assumption. The chosen effect size is β, i.e., the standardized *b* coefficient. Following Cohen (1988), |β| ≥0.10 is considered a small, |β| ≥0.30 a medium, and |β| ≥0.50 a large effect size.

For the qualitative analysis, the interviews were audio-taped and transcribed using open access transcription software and checked by two undergraduate students. Then, an initial deductive coding system was created by the first and second author based on the interview guide and relevant research outlined in the introduction and the study protocol (39), regarding stigma (7), resilience (15), and mental health literacy (64). All of the material was coded using MAXQDA version 2024 by two coders following a deductive-inductive content analysis approach as described by Kuckartz and Rädiker (65): the first and third author first coded individually and then consensually. If a consensus could not be reached, the second and last authors were consulted. The first and the last authors had multiple years of experience in qualitative data analysis.

## 3 Results

The participant flow is depicted in Figure 1, the final sample sizes can be found in the section “Numbers analyzed.”

### 3.1 Quantitative results

#### 3.1.1 Baseline equivalence

The final sample comprises all participants who have provided data for T1 and at least one of the subsequent timepoints (T2 and/or T3).

In terms of sociodemographic composition (Table 2), the intervention group was older than the control group, had lower levels of formal education, and comprised more participants working in university administration. When comparing the baseline data for the sample taking part in T1 and T2 only, the descriptive difference in conservation values was statistically significant, the intervention group holding higher levels of conservation values (Δ*M* = 0.35, *t*(90) = 2.05, *p* = 0.043). Age, on the other hand, did not reach statistical significance in the T1–T2-only sample (Δ*M* = 3.78, *t*(92) = 1.90, *p* = 0.060). There were no significant differences regarding mental distress respectively current or past experiences with personal mental illness or experiences with mental illness due to personal environment or professional background.

**Table 2:** Sociodemographic description of the intervention and control group (baseline values).

Variable	Intervention group			Control group			
	*n*	*M*	*SD*	*n*	*M*	*SD*	*p-*value
Age	54	41.7	9.28	45	37.69	9.76	0.039
Mental distress	53	7.26	4.62	44	7.89	5.14	0.532
PV: self-transcendence	53	5.24	0.45	45	5.20	0.53	0.660
PV: self-enhancement	53	3.58	0.75	45	3.69	0.78	0.484
PV: conservation	53	4.21	0.70	45	4.15	0.74	0.066
PV: openness to change	52	3.76	0.78	45	3.45	0.87	0.656
				**IG** ***n*** **(%)**	**CG** ***n* (%)**		***p*** **-value** ^a^
Gender: female				38 (70.4)	31 (68.9)		0.877
Gender: male				14 (25.9)	11 (24.4)		
Gender: other (e.g., non-binary)				2 (3.8)	3 (6.6)		
Postgraduate education (e.g., Diploma, Master's, Ph.D.)				30 (55.6)	37 (82.2)		0.009
No postgraduate education (e.g., Bachelor, A-levels)				24 (44.4)	8 (17.8)		
Occupation: admin				34 (63.0)	15 (33.3)		0.001^b^
Occupation: research				12 (22.2)	27 (60.0)		
Occupation: other				8 (14.8)	3 (6.7)		

The table shows the baseline data of all participants who have provided data at baseline and at least one of the subsequent timepoints.

PV, personal values; IG, intervention group; CG, control group.

^a^P-values refer to differences in gender, education and occupation between IG and CG.

^b^Participants with an “other” occupation were excluded because this category comprised heterogeneous occupations and was not theoretically relevant for the present analyses. However, Fisher's exact test including the heterogeneous “other” category yielded the same conclusion.

As described above, the groups also differed regarding their baseline scores on several outcome measures (see Supplementary Table S3). Therefore, we controlled for the baseline score of the respective outcome as well as age and personal values of conservation where relevant. Due to subgroup sizes (for many subgroups, *n* ≈ 10) and low overall power for our ANCOVAs (see section 3.1.4), we were not able to include occupation as an additional predictor in our models. Instead, to ensure transparency, we provide a descriptive breakdown of outcomes by time point and occupational group, separately for the intervention and control conditions (Supplementary Table S4).

Given our relatively small sample size, including all potentially relevant sociodemographic confounders in addition to the baseline score would have resulted in further loss of power. Therefore, we followed a parsimonious covariate selection strategy, controlling for the two continuous variables of age and personal values of conservation. Firstly, both variables differed considerably between groups and showed at least small (|*r*| ≥ 0.10) correlations with several of our outcomes of interest. Secondly, this is in line with previous research highlighting the relationship between age and stigma (47, 66) as well as values of conservation and stigma (26). Importantly, covariates were included only in models in which they showed at least small correlations with the respective outcome. Overall, the inclusion of covariates did not alter the statistical significance of findings, with the exception of the main effect for willingness to seek help at T3 (H3), which would have reached statistical significance in the model without covariates (*p* = 0.033), and the exploratory analyses concerning the interaction effect for the VASI at T2, which would not have reached statistical significance in the model without covariates (*p* = 0.051).

Baseline scores and age were centered for all hypotheses, whereas, following the proposal available for ESS21 coding and syntax (67), conservation values were not centered for the main effect hypotheses H1–H5, only for the moderator hypothesis H6.

#### 3.1.2 Numbers analyzed

For T2 comparisons, the final sample comprises an intervention group of *n* = 52 and a control group of *n* = 42, i.e., *N* = 94 participants in total. For T3 comparisons, the final sample is reduced to *n* = 38 in the intervention and *n* = 37 in the control group, i.e., *N* = 75 participants in total. Since only *n* = 1 participant took part in the T2 assessment without having taken part in the intervention, we decided to exclude them from the analyses rather than performing intention-to-treat analyses. Combining employees and managers, we reached the sample size calculated *a priori*: *n* ≥ 25 in both the intervention and the control group (39).

Regarding attrition, we tested whether participants who dropped out at either T2 or T3 differed from participants who did not drop out. We analyzed potential differences on sociodemographic variables (age, gender, education, occupation: administrative vs. scientific staff), baseline personal values, baseline mental distress or baseline personal experience of/contact with mental illness, baseline stigma (OMS-WA and VASI), or baseline mental health literacy levels. No such differences were found, except that participants who dropped out at T3 were more likely to have higher baseline stigma levels [VASI: *B* = 0.40, *SE* = 0.19, *z* = 2.14, *p* = 0.033, *OR* = 1.50, 95% *CI* (1.05, 2.22)]. The second exception was that participants who dropped out at T3 were also more likely to have higher baseline levels of openness values when controlling for other values [PVQ-Openness: *B* = 0.84, *SE* = 0.38, *z* = 2.18, *p* = 0.030, OR = 2.31, 95% *CI* (1.12, 5.12)]. Multicollinearity was assessed and was present to a negligible extent.

#### 3.1.3 Outcomes and estimations

The complete results of the inferential statistical analyses regarding the main (H1–H5) and interaction effects (H6) are summarized in Supplementary Table S5. The model parameters for the complete models and a correlation matrix can also be found in the supplementary material (Supplementary Tables S6, S7).

*(H1) Mental illness stigma:* on the OMS-WA, controlling for values of conservation, the intervention group did not differ significantly from the control group although the effect size was medium [β = 0.31, 95% CI (−0.05, 0.68)]. The other mental illness stigma outcome measures also failed to reach significance, showing small effect sizes [β_SSMIS − agree_ = −0.07, 95% CI (−0.44, 0.31); β_VASI_ = −0.03, 95% CI (−0.32, 0.26); β_SSRPH_ = −0.26, 95% CI (−0.64, 0.13), β_SSOSH_ = −0.17, 95% CI (−0.53, 0.18)].

*(H2) Openness to mental health problems:* controlling for values of conservation, the intervention group did not differ significantly from the control group. The effect size is small [β = 0.24, 95% CI (−0.06, 0.55)].

*(H3) Willingness to seek help:* the intervention group did not differ significantly from the control group. The effect size is small [β = −0.24, 95% CI (−0.55, 0.06)].

*(H4) Resilience:* the intervention group did not differ significantly from the control group. The effect size is small [β = −0.12, 95% CI (−0.42, 0.18)].

*(H5) Mental health literacy:* the intervention group differed significantly from the control group, intervention group participants reporting higher mental health literacy after the intervention. The effect size is large [β = −0.69, 95% CI (−1.07, −0.31)].

*(H6quan) Moderator effect of self-transcendence values:* there was no evidence that self-transcendence values (at T1) moderated the effect of the intervention on any of the mental illness stigma outcomes. For the inferential statistics regarding H6 for T2, see Supplementary Table S6.

Regarding the 6-month follow-up (T3) comparisons, there was no evidence of any statistically significant differences between the intervention and the control group [H1: β_OMS − WA_ =0.15, 95% CI (−0.30, 0.60); β_SSMIS − agree_ = −0.13 (−0.56, 0.30); β_VASI_ =0.03 (−0.35, 0.41); β_SSRPH_ =0.15 (−0.32, 0.62); β_SSOSH_ = −0.13 (−0.55, 0.29); H2: β = 0.06, 95% CI (−0.32, 0.43); H3: β = −0.35, 95% CI (−0.71, 0.01); H4: β = 0.12, 95% CI (−0.21, 0.46); H5: β = −0.40, 95% CI (−0.86, 0.06)]. Of note, there were some medium sized effects which did not reach statistical significance, but are of interest to further program evaluations: firstly, the difference regarding the willingness to seek help (H3), with the intervention group participants reporting higher willingness than control group participants; secondly, the difference in mental health literacy (H5), with the intervention group participants reporting higher mental health literacy than control group participants; thirdly, that of self-transcendence values (H6) moderating the intervention's effect on public help seeking-stigma. For the full inferential statistics, see Supplementary Table S5.

*Post-hoc* power analyses conducted with G^*^Power (68) yielded a power of 0.42 for the statistical test of H1 at T2 with the OMS-WA for employees. Regarding the statistical test of H5, i.e., the effect of group on mental health literacy, at T2, we were able to detect the effect with a power of 0.95 (reduced to 0.42 at T3).

#### 3.1.4 Ancillary analyses

**Figure 2:**
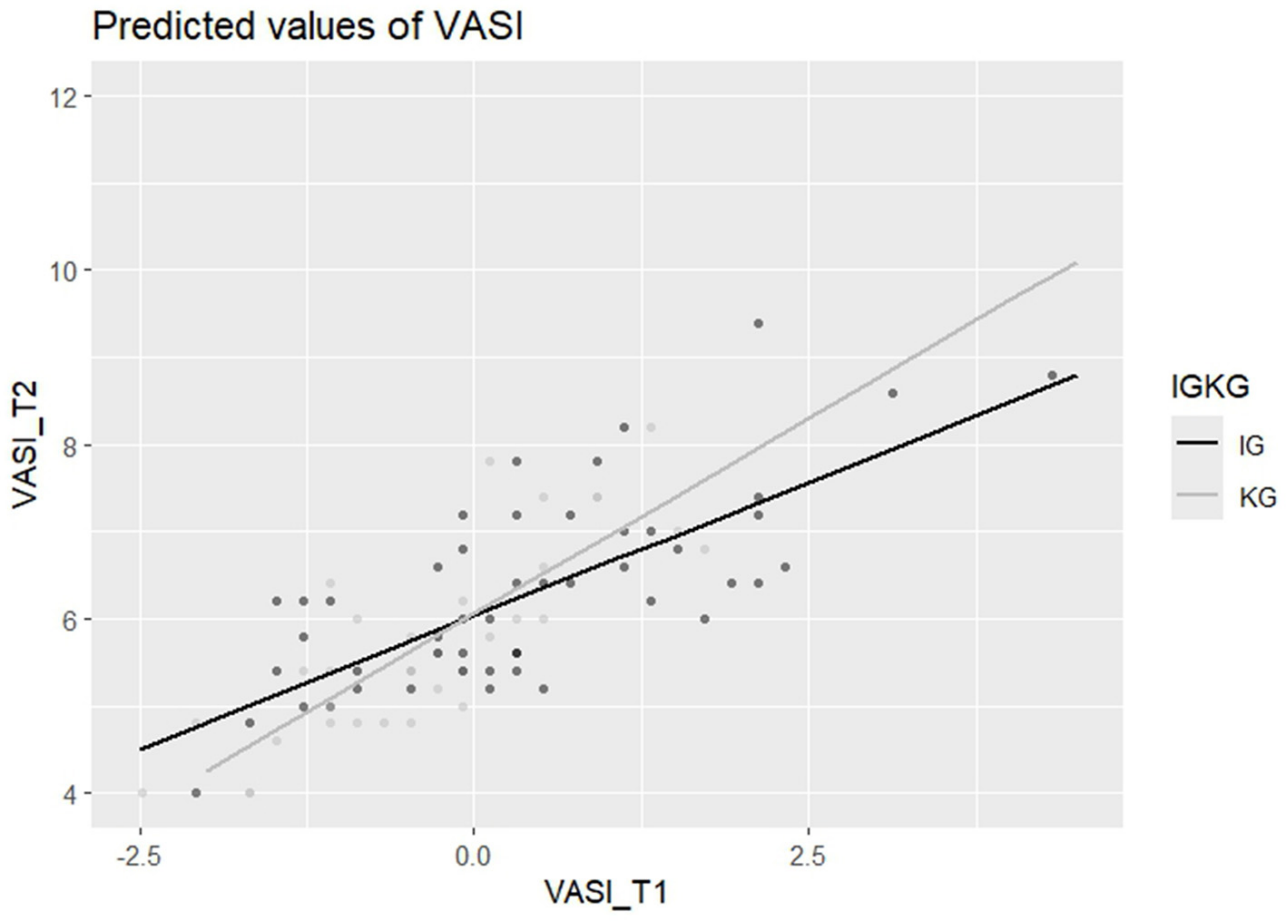
VASI interaction effect at T2, after the workshop.

**Figure 3:**
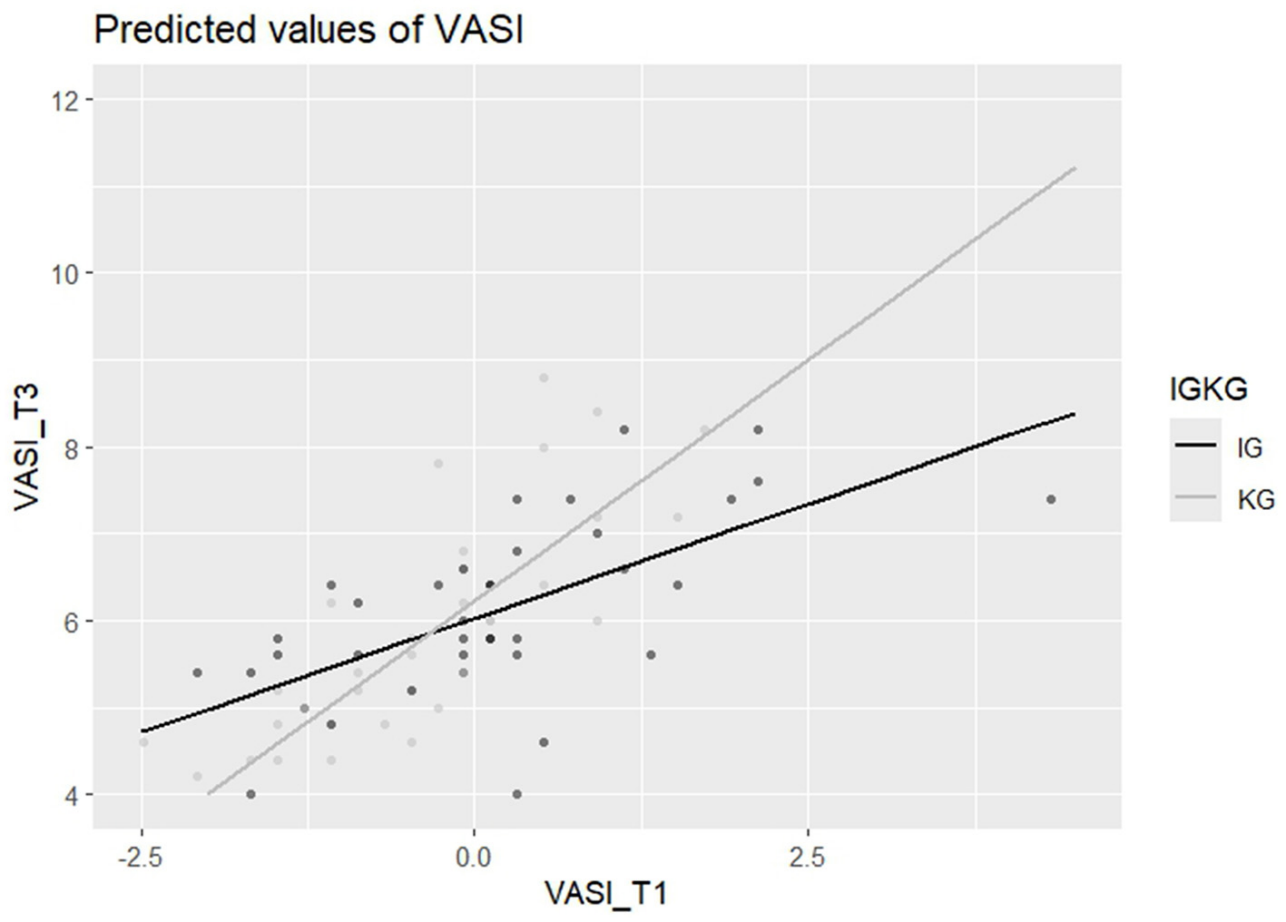
VASI interaction effect at T3, 6-months follow-up.

In addition, we explored for both T2 and T3 whether the effect of the intervention on the outcomes differed according to baseline scores, i.e., testing an interaction between the baseline scores and intervention outcomes (after controlling for the respective covariates of age and values of conservation if they were introduced into the respective models, see description above). None of the interaction effects were significant, except for the VASI (see Figures 2, 3), at both T2 and T3 (T2: *b*_VASI_T1xgroup_ = 0.28, *SE* = 0.14, *t*(81) = 2.00, *p* = 0.049, T3: *b*_VASI_T1xgroup_ = 0.59, *SE* = 0.18, *t*(65) = 3.22, *p* = 0.002). The results thus indicate that the higher the baseline stigma of participants in the intervention group, the lower their stigma values after the intervention compared to participants in the control group with similar baseline stigma values. The size of the interaction effect is medium for T2 [β = 0.31, 95% CI ( 0.00, 0.61)] and large for T3 [β = 0.57, 95% CI (0.22, 0.91)]. The VASI interaction effect at T2 was detected with a power of 0.53. At T3, the power rose to 0.92.

### 3.2 Qualitative results

Seventeen workshop participants took part in the qualitative survey. Participants' characteristics are described in Supplementary Table S8. Overall, the participants in the qualitative part of the study reflect the composition of the participants in the quantitative part of the study with regard to gender and occupation. For the coding process, the intercoder-reliability was excellent, with values of 0.80–0.90 after the consensus process for the core categories (see Supplementary Table S9). In the following section, key findings for each hypothesis will be presented. If relevant, differences between employees and managers will be highlighted. The same hypotheses are investigated, except for “willingness to seek help,” as this quantitative measure was obtained at follow-up and qualitative data was derived ca. 6 weeks after the workshop. The category related to this hypothesis is seeking and offering support or just “support.”

*(H1) Stigma:* participants denied stigmatizing attitudes or behaviors and did not report any experience of discrimination due to mental illness. Nevertheless, participants were aware of having to overcome internalized mental illness stereotypes:

So, you do have obstacles, even for me (.) it took a long time to say, no, I'm going to find a therapist to work through certain things. You must do that for yourself first, [but] especially in my generation, or even older, it's a difficult topic, right? A no-go until, really, do you really need a therapist now? Seriously? (Manager3, pos. 30)

Several participants (five out of 17) were not aware of their stereotyping attitudes, though. They assumed that people living with mental illness could not work in the university environment:

So, if someone is really manifestly mentally ill, they are not here and do not work here. (Employee4, pos. 56)

*(H2) Openness:* overall, participants reported an increased openness toward mental illness, whether it was on an intra- or interpersonal level, talking to other team members about mental issues:

So, I notice that I am in contact with (..) colleagues. That I also/I know from some people here and from other areas who took part in the workshop that we somehow talk about things. So, I have the feeling, so (.) that a different awareness is developing for this. In myself, but also by talking to others who are thinking about it. (Employee4, pos. 102)

Especially for managers the workshop context offered a space for exchange. They valued this a lot, reporting that such spaces were lacking otherwise in their day-to-day work. Not least, in addition to intrapersonal openness, structural openness for mental issues increased because of managers reporting higher personal openness toward mental issues due to the workshop:

Yes, I definitely speak to them earlier, even with my own employees, because you notice that something seems to be wrong here, so you enter into conversation more quickly so that no misunderstandings arise, and through this, [fewer] conflicts evolve. (Manager4, pos. 46)

*(H3) Support:* this core category was coded together with openness, resulting in the subcategories self-help and offering mental health related support to others. Several (seven out of 17) participants were thinking about how to use their knowledge about support offers to help others:

For me, I tend to approach others when I notice that a limit has been reached and that we need to intervene. In the past, I would have ignored it more. Everyone must sort it out for themselves. But now I actively approach them and talk about it. And of course, I also offer my help, what can I do? (.) I signal that I am here. If you need me, you are welcome to come. (Focus group, Speaker06, pos. 68)

Fewer (two out of 17) participants reported that they would use the support offers for themselves; however, a few people realized that they needed help and have started using support offers because of the workshop.

*(H4) Resilience:* participants reported some changes in dealing with stress and adapting to it. Respondents found the knowledge about the continuum of health and illness covered in the workshop to be very helpful. This enabled them to better classify their own mental health, reflect on themselves and thus strengthen their intrapersonal resilience. Participants mentioned that they took breaks more consciously:

So, when I'm off work, I'm off work. I've written nothing more, I've done nothing more, that's it. And that's really good for me. (Employee2, pos. 52)

Managers reported that they have learned how to better deal with their employees' mental illness issues, while also being able to set boundaries for themselves. This may indicate that intrapersonal resilience of managers has improved:

And (.) actually the result for me was, no, it's not your job to help them with their mental illness, but to recognize and accept it and then see how (.) how you can manage it so that the person can still somehow manage their tasks well in their role as an employee. (Manager5, pos. 65)

Finally, it should be noted that relational resilience, e.g., teamwork, social support outside of work (15) was not mentioned as often: we coded four quotes for this category in contrast to 15 quotes regarding personal resilience and 27 quotes regarding organizational resilience. However, employees reported that they exchanged ideas with colleagues to deal with their problems and stress more effectively.

*(H5) Mental health literacy* was addressed by all participants, which often overlapped with openness and support and resilience. Participants reported that they improved their knowledge about mental illness generally, about new ways dealing with own mental issues and mental issues of other people and about their knowledge of support offers.

Some (two out of 10) participants stated that the support offers were not new to them, but most (eight out of 10) participants reported that they had not known about the support offers available at the university and in the region before, both of which were presented in the workshop. In particular, the role of the company doctor became clearer.

Participants said that non-stigmatizing language was a new and helpful content in understanding mental illness. Others emphasized aspects like dealing with suicide or the mental health continuum.

Dealing with mental issues of other people was more often a relevant theme for managers, reflecting on how to recognize mental illness issues in their employees, as outlined in the mental health-illness continuum:

Yes, oh, yes, of course, really paying attention to the fluid transitions, that there are many intermediate stages and also, um, then, um, accordingly, um, it doesn't always have to be the same, that you necessarily offer psychological counseling or something like that straight away, but that you recognize these warning signals a little earlier and maybe even have a conversation then. And, um, yes, don't be inhibited about crossing boundaries in some way, but really offer to talk about what you yourself have noticed. (Manager4, pos. 8)

Knowledge about how to deal with their own mental issues was mentioned by the participants several times. They reported that the mental health continuum tool helped them to self-assess the extent of stress they were experiencing.

I also thought the assessment was really great, and that was something new for me, to see that you can classify it, so that you can find your way around it more easily, to see, okay, here you are [on the mental health continuum], and what you can improve so that it gets better and not worse. (Focus group, S01, pos. 40)

*(H6qual) Personal values:* most participants (16 out of 17) stated the workshop transported self-transcendence (benevolence and universalism), as it aimed at acceptance of people who may be different from oneself and at protecting and caring for others:

Um (...) yes, of course, that includes benevolence. (..) So it's just the exchange and there too you see the people, you help each other. (Employee1, pos. 91)

Um, definitely universalism. (..) So (..) this tolerance toward all people. No matter what phase of life a person is in, um (..) it was emphasized again that it is important, right. If we want to talk about the fact that we really don't want to isolate mental illnesses, and of course we don't want to do that, then of course we really must be tolerant toward every person with their quirks, with their mental illnesses, with their physical illnesses, physical disabilities. (Manager3, pos. 129)

Self-direction, an openness to change value, was also mentioned several times (by eight out 17 participants), aiming at the freedom to determine one's own actions in health. Some participants (nine out of 17) reported the value of security was transported regarding a secure safe place in the workshop and at the workplace.

### 3.3 Triangulation of quantitative and qualitative findings

Overall, most of the quantitative findings were detected with a small or medium effect size and were not significant, possibly due to low power. Qualitative and quantitative findings converge with respect to mental health literacy: the workshop was perceived as helpful by participants with (in our sample overall) low stigmatizing attitudes, as it gave a better understanding of mental illness. The findings also did not differ regarding mental illness stigma: In quantitative results, the effects are small and non-significant with overall low tendency of stigmatizing attitudes. A similar picture is offered by qualitative findings, where we did not record many consciously reported changes regarding the reduction of mental illness stigma attitudes or stereotypes in participants, since most of them described themselves as already open-minded toward mental illness. Nonetheless, we observed a tendency toward potentially unconscious stereotypes toward mentally ill people in the workplace, which participants did not question despite the intervention.

Regarding resilience, openness and willingness to seek support, the two data sources converge to some extent: While quantitative comparisons between the intervention and the control group did not reach statistical significance—possibly due to a lack of power –, small positive effects were found, which are supported by the qualitative data: Participants reported a growth in resilience, openness and willingness to seek support, which negates negative consequences of mental illness stigma. Thus, even populations with low levels of stigma may benefit from the intervention. Participants also reported benefits for their personal and professional resilience and reinforced their self-transcendence, both of which are important resources to successfully cope with psychological stress.

Interestingly, following qualitative inquiry, the use of the mental health continuum, building the heart and the core of the TWM workshop, was identified as helpful by most of the participants in dealing with mental health issues in daily life, which supports the key approach of the intervention and shows its promise.

## 4 Discussion

This study explored the effects of the workshop *The Working Mind* on mental illness stigma, resilience, and mental health literacy in a German university setting using a quasi-experimental, sequential explanative mixed-methods research design while considering personal values. The quantitative analysis showed non-significant results for most of the hypothesized effects, even if the effect for workplace-specific stigma reduction was medium-sized. However, a significantly higher baseline-adjusted level of mental health literacy post- intervention compared to the control group could be detected with a large effect, which decreased to a medium but non-significant effect at follow-up. An interaction effect of baseline values and value-based stigma measure between control and intervention groups was significant, the effect increasing at follow-up. The qualitative analysis showed that employees and managers reported benefits from the workshop with regard to most outcomes analyzed.

### 4.1 Interpretation

The quantitative data suggest that baseline-adjusted mental health literacy is significantly higher post-intervention for workshop participants as compared to the control-group. But we could not detect a significantly lower baseline-adjusted level of mental health stigma post-intervention for workshop participants as compared to the control group. The qualitative findings give hints to possible explanations: participants were already open-minded toward mental illness issues, which could have resulted in low statistical variance regarding mental illness. We cannot rule out the possibility that qualitative data was more prone to social desirability effects and the results here are therefore more positive than in quantitative data.

Furthermore, the lack of significance may also be due to the small sample, and, relatedly, low power. The explanation that the lack of significance is related to such methodological concerns rather than a lack of effectiveness of the workshop appears to be the most salient option when examining effect sizes and significance in our study: Large effects (such as mental health literacy) became significant. For medium effects (workplace-specific mental illness stigma, willingness to seek help or the increase of mental health literacy at T3), the significance level is narrowly missed. Small effects at the end show no significance. However, falling in the medium range, the effect size for workplace-specific mental illness stigma is of clinical significance and is larger than the effects of the other, less context-specific measuring instruments for mental illness stigma. As Tóth et al. (2) noted, the significance of stigma reduction effects could depend on the kind of outcome measurement. In our study, more specific mental health stigma measures such as the VASI (32), exploring value-sensitive mental illness stigma and the OMS-WA (9), exploring workplace-related mental illness stigma, were more sensitive to stigma changes than more general measures (e.g., German version of the Self-Stigma of Mental Illness Scale). This underlines the idea of Yang et al. (19), that the stigma process of mental illness is context-specific, as it threatens “what matters most” for the individual, so the measuring instruments must be context-specific, too.

Qualitative findings suggest that the intervention did not succeed in challenging participants to change their stereotypes of mentally ill people at the workplace. The stereotypes still evident in the qualitative data indicate that the program may not suffice to reduce stereotypical attitudes toward all mental disorders, although it comprises two video testimonials from persons with lived experience of schizophrenia and psychosis, both involved in the academic sector. Maybe there is a low awareness of one's own stereotypes regarding those most stigmatized mental disorders (69) due to low “relatability” (70). In consequence, it may be helpful to incorporate indicators and symptoms of these disorders more clearly into the mental health continuum and case studies in future adaptations.

Moreover, qualitative findings could provide an explanation for the different effect sizes found for openness, support and resilience as compared to mental health knowledge. While the first three show small effect sizes, the effect size for mental health knowledge is large in the quantitative results. One possible explanation could be that the two methodological approaches differed in the way they conceptualized the constructs. In the qualitative results, findings for the category of mental health knowledge were overlapped with findings for the other three construct categories. In the quantitative results, however, there were no intercorrelations between openness, support, resilience and mental health knowledge (see Supplementary Table S7), suggesting that the quantitative instruments strongly distinguished the constructs from one another. As our quantitative question for support offers aimed at detecting changes in intentions and behavior, it differs from “pure” knowledge which is assessed by the mental health knowledge construct. Conversely, the qualitative approach may have emphasized potential areas of overlap between the constructs: Knowledge is the prerequisite for the behavior to be carried out. The same might be true for resilience: It is one thing to know what might improve one's own wellbeing and another to behave persistently in that way.

Research on resilience interventions struggles to understand why some intervention effects fail to reach significance (71). BRS as a generic and brief measure might not be able to mirror the workplace-specific changes that, according to the qualitative data, have occurred due to the workshop. In our qualitative analysis, the newly acquired knowledge of available support has given the managers a feeling of security and competence in helping their employees and offering them support in dealing with their mental health problems. This may in turn lead to employees experiencing greater structural openness, which may then strengthen organizational resilience. Some managers were also able to recognize this connection and were aware of the impact of their actions on the resilience of their employees. According to the findings of Luu et al. (72), the results suggest first changes in institutional culture, local and senior management and harmful behavior, thus maybe improving the university climate.

Finally, there was no moderating effect of self-transcendence values on any measures of mental illness stigma. This might be due to the But, there was a non-significant yet medium sized moderating effect on public help-seeking stigma which is in line with the findings of Lannin et al. (8). Lannin et al. (8) found that self-transcendence values can have an impact on reducing public mental health stigma of seeking help via self-stigma of seeking help. According to the participants in the qualitative study, the workshop emphasized the value of self-transcendence and thus supported the people holding this value, resulting in tendentially lower help seeking stigma (in line with 8). In our sample, in contrast to the cited literature above (24–26), self-transcendence showed no substantial correlations with measures of mental illness stigma, even if higher values were connected to lower stigma (see Supplementary Table S7). The link between conservation values and stigma measures was somewhat more clearly. The significant interaction effect of the VASI after the workshop and at follow-up shows that participants with higher mental illness stigma before the workshop benefited more from the workshop and reported less stigma toward mental illness compared to the control group. The VASI is supposed to correlate negatively with self-transcendence and positively with conservativism (32), which is in line with our findings (see Supplementary Table S7). Last but not least, the results could allow for the formulation of a cautious hypothesis about how values might have had an impact on participants of the workshop. The intervention group participants have shown more pronounced personal values of conservation. In qualitative inquiry, participants mostly attributed self-transcendence values to the workshop, i.e., tolerance and protection for the welfare of all people. In Schwartz Theory of Human Values (20), conservation values are more connected to self-protection and anxiety avoidance, while self-transcendence values are related to growth and overcoming of anxiety. According to cognitive dissonance theory first formulated by Leon Festinger (23, 73), individuals are driven to reduce the negative affects produced by mismatches between their cognitions and the behaviors they perform (73). So the participation in the workshop potentially resulted in more cognitive dissonance for participants holding more conservative values (i.e., striving to adopt to norms and traditions) and thus changed their attitudes toward mental illness, as cognitive dissonance can reduce stigma (23). However, this effect could also work in the other direction, namely that participants with more conservative values did not participate in the study to avoid experiencing cognitive dissonance. Further, cognitive dissonance may be connected to the feeling of responsibility of choosing a behavior, i.e. to high vs. “low choice perception,” the latter is not supposed to induce cognitive dissonance [(74), p. 1]. We can only speculate whether these aspects were relevant for our study participants, but the hypothetical explanation seems worth mentioning and might be further examined empirically.

### 4.2 Limitations and Implications

Since our study is exploratory in nature, our findings should be interpreted with caution. For instance, we did not apply formal corrections for multiple testing since such adjustments would have further decreased power and increased the already elevated risk for Type II error. However, conducting multiple tests without correction increases the probability of Type I error; the results should therefore be considered preliminary. The small sample and the lower number of scientific employees and managers in contrast to non-scientific participants in the intervention group restrict the generalizability of the study to academic staff. We controlled for baseline values between intervention and control group. But there could still be a lack of internal validity as the control group consisted of more scientific and the intervention group of more administrative staff, which we were unable to control for in our analyses due to small subgroups and already low power. Reasons for non-participation among scientific staff in the intervention group are speculative but could be a lower priority of mental health as opposed to scientific output (34, 35, 38) or less hierarchical or supportive structures in their sector. This low interest in participation in TWM in our study is salient at the management level: More administrative managers took part in the study than scientific ones. This could explain the different participation numbers, as some managers of administrative staff recommended the workshop to their employees and they could participate during working hours with compensation. The scientific employees' non-participation in the workshop may be explained by high levels of stress, pressure and a feeling of time constraints due to mostly temporary contracts, but this explanation does not apply to scientific managers: Predominantly, scientific managers hold permanent positions. Stress levels at the management level are likely to be comparable between scientific and administrative staff. Nevertheless, the latter participated in the workshop *and* encouraged their employees to participate. Such participation within departments, encouraged by the managers, might even have changed “what matters most” in this local worlds according to Yang et al. (19) and facilitated program participation. Because scientific staff have predominantly temporary contracts, scientific managers also face high turnover, which could lead to a reduced sense of connection with employees. Last but not least, it might be that the reason for non-participation is higher mental illness stigma as described in the theoretical section (37), as being a “successful scientist” seems incompatible with living with a mental illness (38). From our point of view, this consideration makes it imperative to investigate the stigmatization of mental illness, especially among scientific personnel. This raises the question under what circumstances such a workshop would be easier for scientific participants to attend, or whether the workshop needs significant changes in order to be better received by scientific employees and managers.

Interestingly, participants who dropped out were more likely to have openness values. These values are assigned to freedom of anxiety and growth and have a personal focus in contrast to the social focus of self-transcendence or conservativism values according to Schwartz' value theory (20). We advertised the study in a context of helping others at the workplace, maybe this focus was not interesting enough for participants with more pronounced self-direction and stimulation values.

Furthermore, in line with the literature (75, 76), few men attended our workshops. More informal methods, such as invitations from female participants, could be a better solution to boost male enrolment, which is important, as men are at risk regarding stigmatization of mental illness and associated lower help-seeking behavior (75, 76). Finally, further research is warranted, using randomized controlled trials, possibly in other higher education settings and with a particular emphasis on reaching academic staff and male participants as well as with a look at different outcomes and measures (e.g., workplace-specific resilience, value-based stigma). The losses at follow-up in the workplace-based stigma and resilience measures are in line with the Canadian results and support Dobson et al. (9)' suggestion to offer “booster sessions” fostering sustainable attitudinal change.

## 5 Conclusion

Overall, TWM showed some positive effects in its first adaptation to another cultural and the higher education working context. As our study is the first to evaluate TWM using a control group, our findings underline the positive effects of TWM found in previous studies and seem promising for further program use. Nonetheless, most of our hypothesized effects lacked significance, probably due to a lack of power. Further research should replicate our findings in bigger samples and other different cultural and working contexts, using context-sensitive instruments for measuring stigma.

## Data Availability

The trial was preregistered in the German Clinical Trials Register (trial ID: DRKS00033523) on 31/01/24 and has also been transferred to the WHO registry (main ID: DRKS00033523). The preregistered and peer-reviewed study protocol, study materials, and research data are available at the OSF: https://osf.io/qrjce/?view_only=562269481229499c9467d750b7021e4c.
