# Supplementary material for: Reducing mental health stigma in the workplace: a mixed-method analysis of a quasi-experimental trial and the contextual role of personal values
Source: Front Public Health. 2026 Apr 17;14:1758132. doi: 10.3389/fpubh.2026.1758132 (PMC13133922; doi:10.3389/fpubh.2026.1758132)
Supplement: Supplementary file 1 [file Table_1.docx]

| Section and topic | Content of PPI |
| --- | --- |
| Aim | Employees and managers were involved to better address the cultural adaptation of TWM in a German university context and to understand which changes on the original material should be met to address this special workplace context. |
| Methods | Focus groups with employees and managers were conducted before the workshop to check on the plausibility, usefulness and comprehensibility of the translated and adapted materials. After the workshop interviews and focus groups were conducted to better understand possible changes through the intervention and which improvements should be met for further interventions. |
| Study results | Some changes were met on the original material due to feedback of participants prior to the intervention. After the intervention, participants feedback helped to better understand the results on stigma, resilience and personal values. Furthermore, it helped to identify how to improve the intervention for a university sample. However, male participants were also represented in step two, understanding the results of the intervention in interviews and focus groups, although there was a low number of male participants. Finally, scientific participants were present in both phases, pre- and post-intervention, although they were less represented in the workshops itself. |
| Discussion | PPI helped to design the intervention and understand it results. The overall under-representation of male participants in the intervention did not mirror in the PPI. 24% of the participants in the PPI after the intervention were male, compared to 27% of male intervention group participants. That means, once male participants could be recruited, they also took part in focus groups and interviews with the same possibility as female participants. However, it seems important to involve male participants and participants from other gender into recruiting strategies for intervention, as the research team was predominantly female. |

STable 1: Patient public involvement (PPI) in TWM adaptation and evaluation, GRIPP 2 short form
