# Supplementary material for: Reducing mental health stigma in the workplace: a mixed-method analysis of a quasi-experimental trial and the contextual role of personal values
Source: Front Public Health. 2026 Apr 17;14:1758132. doi: 10.3389/fpubh.2026.1758132 (PMC13133922; doi:10.3389/fpubh.2026.1758132)
Supplement: Supplementary file 2 [file Table_2.pdf]

Supplementary Table 2: Overview of the workshop modules

**Module number and name**	**Module content**
**1 Mental Health and Stigma**	• Basic concepts of mental health and illness<br>- Definitions<br>- Mental Health Continuum<br>- Prevalences<br>• Types and effects of stigma<br>• Knowledge of how to reduce stigma and other barriers in the workplace<br>- Person-centered language<br>- Overcoming barriers for seeking help
**2 Mental Health in the Workplace**	• Basic facts about mental health in the workplace<br>- Occupational health management<br>- Risk factors for mental health<br>• Recognize changes in your own mental health and take appropriate measures<br>- Applications of the Mental Health Continuum Model<br>• How to talk about mental health and illness in the professional and private environment
**3 Stress and Resilience**	• Recognize and practice coping strategies<br>- External and internal stressors<br>- Stress reactions (fight, flight, freeze)<br>- Resilience<br>- Big 4-strategies (deep breathing, positive self-talk, mental replay, goal setting)<br>• Recognize and use available resources to protect yourself and others<br>- Support and help services

**4 Supporting your Team (for managers only)**	• Recognize and use available tools and resources to support the mental health of the team<br>- Having a healthy conversation<br>- Mental Health Continuum Tool for a healthy employees' environment<br>- Dealing with suicidal thoughts<br>- (Return to) work and professional integration<br>- Crisis management
