# Supplementary material for: Reducing mental health stigma in the workplace: a mixed-method analysis of a quasi-experimental trial and the contextual role of personal values
Source: Front Public Health. 2026 Apr 17;14:1758132. doi: 10.3389/fpubh.2026.1758132 (PMC13133922; doi:10.3389/fpubh.2026.1758132)
Supplement: Supplementary file 3 [file Table_3.pdf]

STable 3: Descriptive statistics for the outcome variables by group and time point

Supplementary Table 3: Descriptive statistics for the outcome variables by group and time point

STable 3: Descriptive statistics for the outcome variables by group and time point

	Intervention group			Control group		
**Outcome**	*n*	*M*	*SD*	*n*	*M*	*SD*
			**T1**			
MI stigma: OMS-WA (Employees)	38	1.80	0.33	32	1.56	0.33
MI stigma: SSMIS-agree	53	1.67	0.40	44	1.56	0.58
MI stigma: VASI	53	6.41	1.32	44	5.80	1.19
MI stigma: SSRPH	53	10.32	3.51	44	10.59	2.99
MI stigma: SSOSH	53	22.21	5.80	44	21.09	4.50
Openness to mental health probl. [a]	53	3.93	0.62	43	4.14	0.59
Willingness to seek help	53	2.69	1.11	43	2.49	0.79
Resilience	53	3.40	0.71	45	3.17	0.74
Mental health literacy [a]	53	49.49	10.70	43	53.30	12.19
			**T2**			
MI stigma: OMS-WA (Employees)	37	1.69	0.34	27	1.59	0.36
MI stigma: SSMIS-agree	52	1.57	0.51	38	1.52	0.61
MI stigma: VASI	52	6.23	1.14	37	5.58	1.19
MI stigma: SSRPH	52	10.54	3.00	37	10.24	2.89
MI stigma: SSOSH	51	21.98	5.57	37	20.92	4.98
Openness to mental health probl. [a]	50	3.76	0.51	37	4.07	0.55
Willingness to seek help	51	2.94	1.09	37	2.58	1.03
Resilience	52	3.44	0.64	41	3.22	0.63
Mental health literacy [a]	51	59.49	9.77	37	55.03	10.73
			**T3**			
MI stigma: OMS-WA (Employees)	27	1.78	0.35	29	1.64	0.33
MI stigma: SSMIS-agree	37	1.49	0.40	36	1.47	0.52
MI stigma: VASI	37	6.10	1.03	36	5.72	1.38
MI stigma: SSRPH	37	9.81	3.22	36	10.64	3.55
MI stigma: SSOSH	36	22.44	5.87	36	20.44	5.18
Openness to mental health probl. [a]	37	3.90	0.54	36	4.12	0.55
Willingness to seek help	37	2.95	1.02	36	2.51	0.83
Resilience	38	3.35	0.72	37	3.26	0.86
Mental health literacy [a]	37	57.22	11.02	36	54.25	12.39
			**Utilisation of support offers at T3**			

STable 3: Descriptive statistics for the outcome variables by group and time point

	n yes (%)	*n* no (%)	*n* yes (%)	*n* no (%)
Informal social support	35 (94.6)	2 (2.1)	32 (88.9)	4 (11.1)
Psychosocial counselling	3 (8.1)	34 (91.9)	3 (8.3)	33 (91.7)
Crisis hotline	0 (0.0)	37 (100.0)	1 (2.3)	35 (97.2)
Occupational medical service	1 (2.7)	36 (97.3)	0 (0.0)	36 (100.0)
Specialist doctor	17 (45.9)	20 (54.1)	25 (69.4)	11 (30.6)
Workplace integration management	1 (2.7)	36 (97.3)	1 (2.3)	35 (97.2)

Note. MI stigma = mental illness stigma.

[a] Scale reverse-coded to facilitate interpretation.
