# Supplementary material for: Reducing mental health stigma in the workplace: a mixed-method analysis of a quasi-experimental trial and the contextual role of personal values
Source: Front Public Health. 2026 Apr 17;14:1758132. doi: 10.3389/fpubh.2026.1758132 (PMC13133922; doi:10.3389/fpubh.2026.1758132)
Supplement: Supplementary file 4 [file Table_4.pdf]

Supplement Material Table 4A: Descriptive statistics for the outcome variables by occupation and time point, intervention group participants only

	Administrative staff			Scientific staff		
**Outcome**	*n*	*M*	*SD*	*n*	*M*	*SD*
			**T1**			
MI stigma: OMS-WA (Employees)	24	1.79	0.34	9	1.73	0.33
MI stigma: SSMIS-agree	33	1.70	0.41	12	1.52	0.38
MI stigma: VASI	33	6.62	1.49	12	5.75	0.67
MI stigma: SSRPH	33	10.27	3.79	12	10.33	3.45
MI stigma: SSOSH	22	20.52	4.94	12	24.50	5.23
Openness to mental health probl. [a]	33	3.92	0.59	12	3.99	0.58
Willingness to seek help	33	2.76	1.09	12	2.32	0.89
Resilience	22	3.41	0.81	12	3.31	0.52
Mental health literacy [a]	33	48.09	10.59	12	50.17	12.94
			**T2**			
MI stigma: OMS-WA (Employees)	23	1.68	0.39	9	1.60	0.21
MI stigma: SSMIS-agree	32	1.66	0.49	12	1.40	0.55
MI stigma: VASI	32	6.28	1.22	12	5.97	0.75
MI stigma: SSRPH	32	10.69	3.06	12	10.17	3.49
MI stigma: SSOSH	31	21.48	5.63	12	22.08	4.76
Openness to mental health probl. [a]	31	3.68	0.46	11	3.94	0.47
Willingness to seek help	31	3.15	1.13	12	2.52	1.13
Resilience	32	3.40	0.73	12	3.53	0.41
Mental health literacy [a]	31	58.35	9.90	12	59.92	11.70
			**T3**			
MI stigma: OMS-WA (Employees)	13	1.79	0.37	9	1.66	0.23
MI stigma: SSMIS-agree	21	1.51	0.43	11	1.47	0.36
MI stigma: VASI	21	6.21	1.20	11	5.89	0.87
MI stigma: SSRPH	21	9.76	3.11	11	9.73	3.77
MI stigma: SSOSH	21	21.48	5.96	11	23.36	6.23
Openness to mental health probl. [a]	21	3.79	0.55	11	4.02	0.50
Willingness to seek help	21	3.16	1.11	11	2.68	0.97
Resilience	22	3.40	0.79	11	3.24	0.77
Mental health literacy [a]	21	56.62	10.34	11	57.36	13.62

Note. MI stigma = mental illness stigma. Participants with an "other" occupation were excluded because this category comprised heterogeneous occupations and was not theoretically relevant for the present analyses. [a] Scale reverse-coded to facilitate interpretation.

Supplement Material Table 4B: Descriptive statistics for the outcome variables by occupation and time point, control group participants only

	Administrative staff			Scientifc staff		
**Outcome**	*n*	*M*	*SD*	*n*	*M*	*SD*
		**T1**				
MI stigma: OMS-WA (Employees)	12	1.71	0.40	18	1.44	0.25
MI stigma: SSMIS-agree	14	1.56	0.60	27	1.51	0.54
MI stigma: VASI	14	5.81	1.12	27	5.72	1.25
MI stigma: SSRPH	14	9.64	2.98	27	11.04	2.99
MI stigma: SSOSH	14	20.79	5.44	27	21.41	4.15
Openness to mental health probl. [a]	14	4.01	0.65	26	4.21	0.59
Willingness to seek help	14	2.55	0.86	26	2.51	0.78
Resilience	15	3.17	0.81	27	3.13	0.73
Mental health literacy [a]	14	55.07	10.96	26	52.88	12.56
		**T2**				
MI stigma: OMS-WA (Employees)	10	1.74	0.40	15	1.48	0.29
MI stigma: SSMIS-agree	13	1.48	0.56	22	1.48	0.62
MI stigma: VASI	13	5.69	1.39	21	5.37	0.97
MI stigma: SSRPH	13	10.46	2.54	21	10.38	3.12
MI stigma: SSOSH	13	20.31	6.12	21	21.57	4.53
Openness to mental health probl. [a]	13	3.90	0.60	21	4.15	0.52
Willingness to seek help	13	2.63	0.94	21	2.66	1.12
Resilience	14	3.05	0.63	24	3.28	0.63
Mental health literacy [a]	13	57.15	8.61	21	53.67	11.80
		**T3**				
MI stigma: OMS-WA (Employees)	6	1.73	0.39	20	1.62	0.33
MI stigma: SSMIS-agree	11	1.71	0.63	22	1.36	0.42
MI stigma: VASI	11	6.38	1.60	22	5.35	1.19
MI stigma: SSRPH	11	10.73	4.20	22	10.91	3.41
MI stigma: SSOSH	11	19.27	6.03	22	20.55	4.90
Openness to mental health probl. [a]	11	3.98	0.59	22	4.26	0.49
Willingness to seek help	11	2.46	0.84	22	2.64	0.80
Resilience	11	3.41	0.75	23	3.13	0.93
Mental health literacy [a]	11	55.55	9.46	22	55.14	12.54

Note. MI stigma = mental illness stigma. Participants with an “other” occupation were excluded because this category comprised heterogeneous occupations and was not theoretically relevant for the present analyses. [a] Scale reverse-coded to facilitate interpretation.
