# Supplementary material for: Reducing mental health stigma in the workplace: a mixed-method analysis of a quasi-experimental trial and the contextual role of personal values
Source: Front Public Health. 2026 Apr 17;14:1758132. doi: 10.3389/fpubh.2026.1758132 (PMC13133922; doi:10.3389/fpubh.2026.1758132)
Supplement: Supplementary file 5 [file Table_5.pdf]

STable5: Quantitative results for the hypothesized main (H1-5) and interaction effects (H6)

Supplementary Material Table 5: Quantitative results for the hypothesized main (H1-5) and interaction effects (H6)

STable5: Quantitative results for the hypothesized main (H1-5) and interaction effects (H6)

Outcome	*b*	*SE*	β	95% *CI*	*t*	df_t	*p*
				**T2**			
MI stigma: OMS-WA (Employees) [a]	0.11	0.06	.31	[-.05, .68]	1.72	55	.091†
Interaction self-transcendence [a]	-0.14	0.12	-.19	[-.54, .16]	-1.09	55	.279
MI stigma: SSMIS-agree [a, b]	-0.04	0.10	-.07	[-.44, .31]	-0.34	82	.730
Interaction self-transcendence [a, b]	0.08	0.21	.07	[-.30, .44]	0.38	80	.702
MI stigma: VASI [a]	-0.03	0.17	-.03	[-.32, .26]	-0.02	82	.842
Interaction self-transcendence [a]	-0.16	0.33	-.07	[-.34, .21]	-0.48	80	.635
MI stigma: SSRPH [b]	-0.75	0.56	-.26	[-.64, .13]	-1.34	83	.185
Interaction self-transcendence [b]	0.06	1.13	.00	[-.37, .39]	0.05	81	.960
MI stigma: SSOSH [a, b]	-0.92	0.94	-.17	[-.53, .18]	-0.98	80	.332
Interaction self-transcendence [a, b]	2.22	1.86	.21	[-.14, .55]	1.19	78	.236
Openness to mental health probl. [a, c]	0.14	0.08	.24	[-.06, .55]	1.60	80	.112
Willingness to seek help	-0.26	0.16	-.24	[-.55, .06]	-1.59	83	.115
Resilience	-0.08	0.10	-.12	[-.42, .18]	-0.80	89	.422
Mental health literacy [c]	-7.14	1.98	-.69	[-1.07, -.31]	-3.61	83	.001**
				**T3**			
MI stigma: OMS-WA (Employees) [a]	0.05	0.08	.15	[-.30, .60]	0.67	47	.511
Interaction self-transcendence [a]	-0.15	0.20	-.16	[-.60, .28]	-0.75	45	.456
MI stigma: SSMIS-agree [a, b]	-0.06	0.10	-.13	[-.56, .30]	-0.59	68	.555
Interaction self-transcendence [a, b]	-0.01	0.19	-.01	[-.43, .41]	-0.06	66	.956
MI stigma: VASI [a, b]	0.03	0.23	.03	[-.35, .41]	0.14	68	.887
Interaction self-transcendence [a, b]	-0.33	0.55	-.13	[-.49, .23]	-0.61	66	.546
MI stigma: SSRPH [b]	0.50	0.80	.15	[-.32, .62]	0.63	69	.534
Interaction self-transcendence [b]	-3.02	1.70	-.44	[-.93, .06]	-1.77	67	.081†
MI stigma: SSOSH [a, b]	-0.74	1.18	-.13	[-.55, .29]	-0.63	67	.532
Interaction self-transcendence [a, b]	-1.92	2.25	-.17	[-.57, .23]	-0.86	65	.395
Openness to mental health probl. [a, b, c]	0.03	0.10	.06	[-.32, .43]	0.30	68	.765
Willingness to seek help [b]	-0.33	0.17	-.35	[-.71, .01]	-1.92	69	.060†
Resilience [b]	0.10	0.13	.12	[-.21, .46]	0.74	71	.462
Mental health literacy [c]	-4.71	2.70	-.40	[-.86, .06]	-1.75	70	.085†
				**Utilisation of support offers at T3**			
	B	*SE*	*OR*	95% *CI*	z		*p*
Informal social support			0.46	[0.04, 3.48]			.430
Psychosocial counselling			1.03	[0.13, 8.25]			1.000

STable5: Quantitative results for the hypothesized main (H1-5) and interaction effects (H6)

Crisis hotline			*Inf* [d]	[0.03, *Inf* [d]]		.493
Occupational medical service			0	[0.00, 40.83]		1.000
Specialist doctor [b]	-0.77	0.51	0.46	[0.17, 1.24]	-1.53	.126
Workplace integration management			1.03	[0.01, 80.90]		1.000

Note. MI stigma = mental illness stigma. All hypotheses except for hypothesis H3 regarding the use of support offers were tested using ANCOVAs. The intervention group constitutes the ANCOVA reference category, i.e., positive values for *b* imply that control group participants score higher on the respective outcome measure, whereas the opposite is true for negative values of *b*. All *b*s are adjusted for baseline differences on the respective outcome measure. Hypothesis H3 regarding the use of support offers at T3 was tested using logistic regression if each of the cell sizes encompassed $n \geq 5$ (specialist doctor); otherwise, Fisher's exact test was used (all other offers).

[a] After controlling for values of conservation. [b] After controlling for age. [c] Scale reverse-ecoded to facilitate interpretation. [d] *Inf* indicates an infinite odds ratio due to a zero cell count in one group.

† $p < .10$. * $p < .05$. ** $p < .01$.
