# Supplementary material for: Reducing mental health stigma in the workplace: a mixed-method analysis of a quasi-experimental trial and the contextual role of personal values
Source: Front Public Health. 2026 Apr 17;14:1758132. doi: 10.3389/fpubh.2026.1758132 (PMC13133922; doi:10.3389/fpubh.2026.1758132)
Supplement: Supplementary file 6 [file Table_6.pdf]

Manuscript:
Reducing Mental Health Stigma in the Workplace: a mixed-method Analysis of a quasi-experimental Trial and the Role of Personal Values

STable 6: Complete T2 and T3 models

Supplement Material Table 6a: Complete T2 models

	**MI stigma: OMS-WA (Employees)**				
Predictor	*b*	*SE*	*95 % CI*	*t*	*p*
(Intercept)	1.47	0.14	1.19 – 1.75	10.57	**<0.001**
IGKG [KG]	0.11	0.06	-0.02 – 0.24	1.72	0.091
OMS WA Besch T1	0.75	0.09	0.58 – 0.92	8.81	**<0.001**
PVQ Cons T1	0.04	0.04	-0.03 – 0.11	1.08	0.285
Observations	61				
R^2 / R^2 adjusted	0.597 / 0.576				

	**MI stigma: OMS-WA (Employees) x self-transcendence**				
Predictor	*b*	*SE*	*95 % CI*	*t*	*p*
(Intercept)	1.60	0.04	1.52 – 1.68	39.49	**<0.001**
IGKG [KG]	0.13	0.07	-0.00 – 0.26	1.95	0.056
PVQ STran T1 c	0.12	0.09	-0.06 – 0.31	1.31	0.195
OMS WA Besch T1	0.79	0.09	0.60 – 0.97	8.60	**<0.001**
PVQ Cons T1 c	0.04	0.04	-0.03 – 0.12	1.16	0.251
IGKG [KG] × PVQ STran T1 c	-0.14	0.12	-0.39 – 0.11	-1.09	0.279
Observations	61				
R^2 / R^2 adjusted	0.610 / 0.574				

	**MI stigma: SSMIS-agree**				
Predictor	*b*	*SE*	*95 % CI*	*t*	*p*
(Intercept)	1.42	0.24	0.94 – 1.90	5.92	**<0.001**
IGKG [KG]	-0.04	0.10	-0.24 – 0.17	-0.35	0.730

SSMIS agree T1	0.62	0.10	0.42 – 0.82	6.16	**<0.001**
PVQ Cons T1	0.04	0.06	-0.08 – 0.16	0.61	0.542
age T1 c	-0.01	0.01	-0.02 – 0.00	-1.90	0.061
Observations	87				
R^2 / R^2 adjusted	0.347 / 0.315				

	**MI stigma: SSMIS-agree x self-transcendence**				
Predictor	*b*	*SE*	*95 % CI*	*t*	*p*
(Intercept)	1.56	0.07	1.42 – 1.69	23.54	**<0.001**
IGKG [KG]	-0.03	0.10	-0.24 – 0.17	-0.33	0.740
PVQ STran T1 c	-0.02	0.15	-0.31 – 0.27	-0.16	0.870
SSMIS agree T1	0.62	0.10	0.42 – 0.83	6.04	**<0.001**
PVQ Cons T1 c	0.03	0.06	-0.10 – 0.16	0.51	0.614
age T1 c	-0.01	0.01	-0.02 – 0.00	-1.77	0.080
IGKG [KG] × PVQ STran T1 c	0.08	0.21	-0.34 – 0.50	0.38	0.702
Observations	87				
R^2 / R^2 adjusted	0.348 / 0.300				

	**MI stigma: VASI**				
Predictor	*b*	*SE*	*95 % CI*	*t*	*p*
(Intercept)	5.44	0.39	4.67 – 6.21	13.99	**<0.001**
IGKG [KG]	-0.03	0.17	-0.37 – 0.30	-0.20	0.842
VASI T1	0.70	0.07	0.56 – 0.83	10.44	**<0.001**
PVQ Cons T1	0.16	0.10	-0.04 – 0.36	1.55	0.126
Observations	86				
R^2 / R^2 adjusted	0.621 / 0.607				

	**MI stigma: VASI x self-transcendence**				
Predictor	*b*	*SE*	*95 % CI*	*t*	*p*

(Intercept)	6.00	0.11	5.79 – 6.22	55.41	**<0.001**
IGKG [KG]	-0.04	0.17	-0.38 – 0.30	-0.22	0.824
PVQ STran T1 c	-0.02	0.24	-0.49 – 0.46	-0.06	0.950
VASI T1	0.69	0.07	0.55 – 0.83	9.92	**<0.001**
PVQ Cons T1 c	0.18	0.11	-0.03 – 0.39	1.67	0.099
IGKG [KG] × PVQ STran T1 c	-0.16	0.33	-0.82 – 0.50	-0.48	0.635
Observations	86				
R^2 / R^2 adjusted	0.624 / 0.600				

	**MI stigma: SSRPH**				
Predictor	*b*	*SE*	*95 % CI*	*t*	*p*
(Intercept)	10.71	0.36	10.00 – 11.42	30.04	**<0.001**
IGKG [KG]	-0.75	0.56	-1.87 – 0.37	-1.34	0.185
SSRPH T1	0.39	0.09	0.23 – 0.56	4.64	**<0.001**
age T1 c	-0.08	0.03	-0.14 – -0.02	-2.76	**0.007**
Observations	87				
R^2 / R^2 adjusted	0.271 / 0.245				

	**MI stigma: SSRPH x self-transcendence**				
Predictor	*b*	*SE*	*95 % CI*	*t*	*p*
(Intercept)	10.74	0.36	10.03 – 11.44	30.24	**<0.001**
IGKG [KG]	-0.81	0.56	-1.92 – 0.31	-1.44	0.155
PVQ STran T1 c	-0.98	0.80	-2.58 – 0.61	-1.22	0.225
SSRPH T1	0.37	0.09	0.19 – 0.54	4.17	**<0.001**
age T1 c	-0.09	0.03	-0.15 – -0.03	-2.96	**0.004**
IGKG [KG] × PVQ STran T1 c	0.06	1.13	-2.19 – 2.30	0.05	0.960
Observations	87				
R^2 / R^2 adjusted	0.296 / 0.253				

	MI stigma: SSOSH				
Predictor	*b*	*SE*	*95 % CI*	*t*	*p*
(Intercept)	19.87	2.19	15.51 – 24.22	9.07	**<0.001**
IGKG [KG]	-0.92	0.94	-2.80 – 0.96	-0.98	0.332
SSOSH T1	0.58	0.09	0.41 – 0.75	6.77	**<0.001**
age T1 c	-0.08	0.05	-0.18 – 0.02	-1.57	0.119
PVQ Cons T1	0.55	0.56	-0.58 – 1.67	0.97	0.335
Observations	85				
R^2 / R^2 adjusted	0.431 / 0.402				

	MI stigma: SSOSH x self-transcendence				
Predictor	*b*	*SE*	*95 % CI*	*t*	*p*
(Intercept)	21.92	0.60	20.73 – 23.11	36.67	**<0.001**
IGKG [KG]	-0.98	0.94	-2.85 – 0.90	-1.04	0.303
PVQ STran T1 c	-2.09	1.32	-4.72 – 0.54	-1.58	0.118
SSOSH T1	0.58	0.09	0.41 – 0.75	6.76	**<0.001**
age T1 c	-0.07	0.05	-0.17 – 0.02	-1.50	0.139
PVQ Cons T1 c	0.60	0.58	-0.56 – 1.75	1.03	0.304
IGKG [KG] × PVQ STran T1 c	2.22	1.86	-1.48 – 5.91	1.19	0.236
Observations	85				
R^2 / R^2 adjusted	0.448 / 0.406				

	Openness to mental health probl.				
Predictor	*b*	*SE*	*95 % CI*	*t*	*p*
(Intercept)	3.95	0.20	3.56 – 4.33	20.15	**<0.001**
IGKG [KG]	0.14	0.08	-0.03 – 0.30	1.61	0.112
psy open T1	0.63	0.07	0.50 – 0.77	9.38	**<0.001**
PVQ Cons T1	-0.02	0.05	-0.12 – 0.08	-0.43	0.672
Observations	84				
R^2 / R^2 adjusted	0.567 / 0.551				

			Willingness to seek help		
Predictor	*b*	*SE*	*95 % CI*	*t*	*p*
(Intercept)	2.89	0.11	2.68 – 3.10	27.34	**<0.001**
IGKG [KG]	-0.26	0.16	-0.58 – 0.06	-1.59	0.115
use intent T1	0.76	0.08	0.60 – 0.93	9.34	**<0.001**
Observations	86				
R^2 / R^2 adjusted	0.525 / 0.513				

			Resilience		
Predictor	*b*	*SE*	*95 % CI*	*t*	*p*
(Intercept)	3.38	0.06	3.25 – 3.51	52.36	**<0.001**
IGKG [KG]	-0.08	0.10	-0.27 – 0.12	-0.81	0.422
BRS T1	0.60	0.07	0.47 – 0.73	9.16	**<0.001**
Observations	92				
R^2 / R^2 adjusted	0.498 / 0.487				

			Mental health literacy		
Predictor	*b*	*SE*	*95 % CI*	*t*	*p*
(Intercept)	60.45	1.26	57.93 – 62.96	47.84	**<0.001**
IGKG [KG]	-7.14	1.98	-11.08 – -3.21	-3.61	**0.001**
MHL W G T1	0.46	0.09	0.28 – 0.63	5.24	**<0.001**
Observations	86				
R^2 / R^2 adjusted	0.289 / 0.272				

Supplement Material Table 6b: Complete T3 models

	MI stigma: OMS-WA (Employees)				
Predictor	*b*	*SE*	*95 % CI*	*t*	*p*
(Intercept)	1.42	0.18	1.07 – 1.77	8.06	**<0.001**
IGKG [KG]	0.05	0.08	-0.11 – 0.21	0.66	0.510
OMS WA Besch T1	0.70	0.11	0.49 – 0.92	6.51	**<0.001**
PVQ Cons T1	0.08	0.04	-0.01 – 0.16	1.71	0.094
Observations	51				
R^2 / R^2 adjusted	0.533 / 0.503				

	MI stigma: OMS-WA (Employees) x self-transcendence				
Predictor	*b*	*SE*	*95 % CI*	*t*	*p*
(Intercept)	1.67	0.05	1.56 – 1.78	30.92	**<0.001**
IGKG [KG]	0.08	0.09	-0.09 – 0.25	0.96	0.342
PVQ STran T1 c	0.18	0.14	-0.11 – 0.47	1.25	0.218
OMS WA Besch T1	0.75	0.11	0.52 – 0.98	6.55	**<0.001**
PVQ Cons T1 c	0.08	0.05	-0.02 – 0.17	1.55	0.129
IGKG [KG] × PVQ STran T1 c	-0.15	0.20	-0.56 – 0.26	-0.75	0.456
Observations	51				
R^2 / R^2 adjusted	0.549 / 0.499				

	MI stigma: SSMIS-agree				
Predictor	*b*	*SE*	*95 % CI*	*t*	*p*
(Intercept)	1.33	0.23	0.86 – 1.80	5.67	**<0.001**
IGKG [KG]	-0.06	0.10	-0.26 – 0.14	-0.59	0.555
SSMIS agree T1	0.40	0.09	0.21 – 0.59	4.30	**<0.001**
PVQ Cons T1	0.05	0.06	-0.06 – 0.17	0.95	0.345
age T1 c	-0.02	0.00	-0.03 – -0.01	-3.18	**0.002**
Observations	73				

R^2 / R^2 adjusted	0.346 / 0.307

	MI stigma: SSMIS-agree x self-transcendence				
Predictor	*b*	*SE*	*95 % CI*	*t*	*p*
(Intercept)	1.54	0.07	1.40 – 1.67	23.16	**<0.001**
IGKG [KG]	-0.07	0.10	-0.27 – 0.12	-0.76	0.452
PVQ STran T1 c	-0.17	0.15	-0.47 – 0.13	-1.10	0.274
SSMIS agree T1	0.37	0.10	0.18 – 0.56	3.93	**<0.001**
PVQ Cons T1 c	0.08	0.06	-0.04 – 0.20	1.39	0.168
age T1 c	-0.02	0.01	-0.03 – -0.01	-3.42	**0.001**
IGKG [KG] × PVQ STran T1 c	-0.01	0.19	-0.40 – 0.38	-0.06	0.956
Observations	73				
R^2 / R^2 adjusted	0.376 / 0.319				

	MI stigma: VASI				
Predictor	*b*	*SE*	*95 % CI*	*t*	*p*
(Intercept)	6.58	0.56	5.46 – 7.69	11.77	**<0.001**
IGKG [KG]	0.03	0.23	-0.43 – 0.50	0.14	0.886
VASI T1	0.75	0.09	0.56 – 0.94	7.90	**<0.001**
PVQ Cons T1	-0.16	0.14	-0.43 – 0.12	-1.12	0.265
age T1 c	0.01	0.01	-0.02 – 0.03	0.69	0.495
Observations	73				
R^2 / R^2 adjusted	0.498 / 0.468				

	MI stigma: VASI x self-transcendence				
Predictor	*b*	*SE*	*95 % CI*	*t*	*p*
(Intercept)	6.02	0.16	5.71 – 6.33	38.79	**<0.001**
IGKG [KG]	-0.00	0.23	-0.47 – 0.47	-0.01	0.993
PVQ STran T1 c	-0.13	0.36	-0.86 – 0.59	-0.37	0.712

VASI T1	0.71	0.10	0.51 – 0.91	7.18	**<0.001**
PVQ Cons T1 c	-0.08	0.15	-0.38 – 0.21	-0.58	0.566
age T1 c	0.00	0.01	-0.02 – 0.03	0.41	0.686
IGKG [KG] × PVQ STran T1 c	-0.33	0.45	-1.22 – 0.56	-0.74	0.462
Observations	73				
R^2 / R^2 adjusted	0.517 / 0.473				

	**MI stigma: SSRPH**				
Predictor	*b*	*SE*	*95 % CI*	*t*	*p*
(Intercept)	10.04	0.55	8.94 – 11.13	18.26	**<0.001**
IGKG [KG]	0.50	0.80	-1.09 – 2.09	0.63	0.533
SSRPH T1	0.27	0.12	0.03 – 0.50	2.27	**0.027**
age T1 c	-0.05	0.04	-0.13 – 0.03	-1.17	0.244
Observations	73				
R^2 / R^2 adjusted	0.105 / 0.067				

	**MI stigma: SSRPH x self-transcendence**				
Predictor	*b*	*SE*	*95 % CI*	*t*	*p*
(Intercept)	10.03	0.55	8.93 – 11.12	18.32	**<0.001**
IGKG [KG]	0.38	0.79	-1.21 – 1.96	0.47	0.637
PVQ STran T1 c	0.91	1.37	-1.83 – 3.65	0.66	0.510
SSRPH T1	0.30	0.12	0.06 – 0.55	2.44	**0.017**
age T1 c	-0.07	0.04	-0.15 – 0.02	-1.62	0.110
IGKG [KG] × PVQ STran T1 c	-3.02	1.70	-6.43 – 0.38	-1.77	0.081
Observations	73				
R^2 / R^2 adjusted	0.166 / 0.104				

	**MI stigma: SSOSH**				
Predictor	*b*	*SE*	*95 % CI*	*t*	*p*

(Intercept)	17.91	2.74	12.44 – 23.38	6.54	**<0.001**
IGKG [KG]	-0.74	1.18	-3.10 – 1.61	-0.63	0.532
SSOSH T1	0.60	0.11	0.39 – 0.81	5.71	**<0.001**
age T1 c	-0.04	0.06	-0.16 – 0.08	-0.62	0.539
PVQ Cons T1	1.15	0.68	-0.20 – 2.50	1.70	0.094
Observations	72				
R^2 / R^2 adjusted	0.398 / 0.362				

	**MI stigma: SSOSH x self-transcendence**				
Predictor	*b*	*SE*	*95 % CI*	*t*	*p*
(Intercept)	22.06	0.78	20.50 – 23.63	28.16	**<0.001**
IGKG [KG]	-0.87	1.18	-3.22 – 1.47	-0.74	0.460
PVQ STran T1 c	-0.55	1.78	-4.11 – 3.01	-0.31	0.759
SSOSH T1	0.58	0.11	0.37 – 0.79	5.54	**<0.001**
age T1 c	-0.06	0.06	-0.18 – 0.06	-0.96	0.340
PVQ Cons T1 c	1.44	0.69	0.06 – 2.83	2.08	**0.041**
IGKG [KG] × PVQ STran T1 c	-1.92	2.25	-6.41 – 2.56	-0.86	0.395
Observations	72				
R^2 / R^2 adjusted	0.425 / 0.372				

	**Openness to mental health probl.**				
Predictor	*b*	*SE*	*95 % CI*	*t*	*p*
(Intercept)	4.28	0.25	3.79 – 4.78	17.17	**<0.001**
IGKG [KG]	0.03	0.10	-0.18 – 0.24	0.30	0.765
psy open T1	0.62	0.08	0.45 – 0.79	7.45	**<0.001**
PVQ Cons T1	-0.09	0.06	-0.21 – 0.03	-1.48	0.144
age T1 c	-0.01	0.01	-0.02 – 0.00	-1.27	0.210
Observations	73				
R^2 / R^2 adjusted	0.502 / 0.473				

	Willingness to seek help				
Predictor	*b*	*SE*	*95 % CI*	*t*	*p*
(Intercept)	2.91	0.12	2.67 – 3.14	24.60	**<0.001**
IGKG [KG]	-0.33	0.17	-0.67 – 0.01	-1.92	0.060
use intent T1	0.65	0.09	0.47 – 0.83	7.29	**<0.001**
age T1 c	0.01	0.01	-0.01 – 0.02	0.59	0.556
Observations	73				
R^2 / R^2 adjusted	0.472 / 0.449				

	Utilisation of specialist doctor					
Predictor	*B*	*SE*	*OR*	*95 % CI*	*z*	*p*
(Intercept)	0.06	0.34	1.06	0.54 – 2.08	0.17	0.865
IGKG [KG]	-0.77	0.51	0.46	0.17 – 1.24	-1.53	0.126
age T1 c	0.05	0.03	1.05	0.99 – 1.11	1.70	0.090
Observations	73					
R^2 Tjur	0.093					

	Resilience				
Predictor	*b*	*SE*	*95 % CI*	*t*	*p*
(Intercept)	3.24	0.09	3.06 – 3.41	35.90	**<0.001**
IGKG [KG]	0.10	0.13	-0.16 – 0.36	0.74	0.462
BRS T1	0.81	0.09	0.63 – 0.99	9.15	**<0.001**
age T1 c	0.01	0.01	-0.01 – 0.02	1.11	0.270
Observations	75				
R^2 / R^2 adjusted	0.550 / 0.531				

	Mental health literacy				
Predictor	*b*	*SE*	*95 % CI*	*t*	*p*
(Intercept)	57.93	1.86	54.22 – 61.64	31.17	**<0.001**
IGKG [KG]	-4.71	2.70	-10.08 – 0.67	-1.75	0.085

MHL W G T1	0.32	0.11	0.09 – 0.55	2.78	**0.007**
Observations	73				
R^2 / R^2 adjusted	0.114 / 0.089				
