# Supplementary material for: Reducing mental health stigma in the workplace: a mixed-method analysis of a quasi-experimental trial and the contextual role of personal values
Source: Front Public Health. 2026 Apr 17;14:1758132. doi: 10.3389/fpubh.2026.1758132 (PMC13133922; doi:10.3389/fpubh.2026.1758132)
Supplement: Supplementary file 7 [file Table_7.pdf]

Supplementary Material Table 7 (STable7): Pearson correlations among the outcome variables, covariates, and moderator variable

Variable	1	2	3	4	5	6	7	8	9	10	11
1. Age [a]											
2. Conservation values [a]	-.09										
3. Self-transcendence values [a]	-.14	.21									
4. MI stigma: OMS-WA (Employees)	-.09	.20	-.11								
5. MI stigma: SSMIS-agree	-.22	.13	-.01	.54							
6. MI stigma: VASI	.03	.28	-.12	.65	.57						
7. MI stigma: SSRPH	-.30	.02	-.19	.29	.27	.19					
8. MI stigma: SSOSH	-.22	.21	-.02	.33	.16	.29	.33				
9. Openness to mental health probl.	-.08	-.18	.26	-.27	-.27	-.44	-.30	-.24			
10. Willingness to seek help	.08	-.01	.08	-.11	.09	-.04	.02	-.19	.04		
11. Resilience	-.07	.02	.07	.05	.12	.19	-.04	.01	-.05	.07	
12. Mental health literacy	-.01	-.00	.15	.10	-.04	-.04	-.12	-.02	.09	.05	.16

Note. Outcome variable measured at T2, covariates and moderator variable measured at T1, consistent with our main analyses.
[a] Covariate/moderator variable.
