# Supplementary material for: Reducing mental health stigma in the workplace: a mixed-method analysis of a quasi-experimental trial and the contextual role of personal values
Source: Front Public Health. 2026 Apr 17;14:1758132. doi: 10.3389/fpubh.2026.1758132 (PMC13133922; doi:10.3389/fpubh.2026.1758132)
Supplement: Supplementary file 8 [file Table_8.docx]

**Supplementary Material Table 8 (STable8): Characteristics of participants for qualitative results**

|  | **Focus Group** | **Interviews** |
| --- | --- | --- |
| **Number of participants** | eight | nine |
| **Gender** | seven female, one male | six female, three male |
| **Professional position** | all employees | five managers, four employees |
| **Professional field of activity** | five in administration,  three in science | six from administration, one from science, two from other university sectors |
